# Interactive Tree Analysis Identifies Dietary Fiber and Magnesium Adequacy as Exploratory Screening Markers for Assessing Nutrient-Dense, Immune-Supportive and Anti-Inflammatory Dietary Patterns in Young Adults Without Comorbidities: Proposition of the New StrongPOLA and RapidPOLA Indexes

**DOI:** 10.3390/nu18111689

**Published:** 2026-05-25

**Authors:** Paweł Jagielski, Philip C. Calder, Izabela Bolesławska, Edyta Łuszczki

**Affiliations:** 1 Department of Nutrition and Drug Research, Institute of Public Health, Faculty of Health Sciences, Jagiellonian University Medical College, 31-066 Kraków, Poland; 2 Faculty of Medicine, NIHR Southampton Biomedical Research Centre, University of Southampton and University Hospital Southampton NHS Foundation Trust, Southampton SO16 6YD, UK; p.c.calder@soton.ac.uk; 3 Department of Bromatology, Poznan University of Medical Sciences, 60-354 Poznań, Poland; ibolesla@ump.edu.pl; 4 Faculty of Health Sciences and Psychology, Collegium Medicum, University of Rzeszów, 35-959 Rzeszów, Poland; eluszczki@ur.edu.pl

**Keywords:** dietary fiber, magnesium, nutrition, POLA index, RapidPOLA, StrongPOLA, immune-supportive diet, NUTRIDIMAF diet, COVID-19, influenza, fiber in g per kg/m^2^ of BMI

## Abstract

**Background/Objectives:** The POLA index is a comprehensive tool for evaluating the nutrient-dense, immune-supportive, and anti-inflammatory properties of the diet, but its multi-component structure may limit routine use. We aimed to identify simple dietary markers associated with a lower follow-up incidence of COVID-19 or influenza, as well as the anti-inflammatory properties of the diet, and to compare a simplified screening tool with the full POLA index. **Methods:** This prospective observational study included 146 healthy adults aged 25–45 years from two Polish cohorts examined in 2020 and 2022 (cohort/year adjusted). Habitual diet was assessed using at least 5-day food records, and nutrient adequacy was expressed relative to Polish dietary reference values. Classification and regression tree analyses were used to identify the most informative dietary predictors of the reduction in risk of infection, and logistic regression models were used to evaluate associations after adjustment for sex, diet type, physical activity, marital status, year of cohort and waist-to-height ratio. **Results:** During follow-up, 39/146 participants (26.7%) reported COVID-19 or influenza. Interactive tree analysis identified dietary fiber in g per kg/m^2^ of BMI ≥ 1, and magnesium adequacy as the key discriminators. In StrongPOLA, participants not meeting the cut-offs of ≥1 g fiber per kg/m^2^ of BMI and ≥130% of the magnesium reference value had a higher incidence of COVID-19 or influenza than those meeting both of those cut-offs (34.9% vs. 2.7%); however, this estimate was large and imprecise, with a wide confidence interval (the adjusted OR = 14.9 (95% CI: 1.89–118.06)), and should, therefore, be interpreted cautiously. In RapidPOLA, the participants not meeting the cut-offs of ≥1 g fiber per kg/m^2^ of BMI and ≥110% of the magnesium reference value (i.e., 352 mg/day for women and 462 mg/day for men) had a higher observed incidence of COVID-19 or influenza than those meeting both of those cut-offs (36.4% vs. 12.1%); the adjusted OR was 3.4 (95% CI: 1.18–8.75). RapidPOLA showed good agreement with the favorable result of the POLA classification (κ = 0.65). **Conclusions:** Dietary fiber in g per kg/m^2^ of BMI and magnesium adequacy appear to be practical markers of a broader nutrient-dense, immune-supporting, and anti-inflammatory dietary pattern associated with a lower follow-up incidence of COVID-19 or influenza in young adults without comorbidities. RapidPOLA may be useful as a simple screening tool for a nutrient-dense, immune-supportive, and anti-inflammatory (NUTRIDIMAF) diet in young people without obesity and comorbidities, whereas StrongPOLA may serve as a stricter reference profile. The proposed cut-offs require external validation in independent and more diverse cohorts.

## 1. Introduction

Over the past six years, a growing body of observational evidence has suggested that healthier dietary patterns, particularly those richer in plant foods, are associated with a lower susceptibility to SARS-CoV-2 infection and/or more favorable COVID-19 outcomes. For example, in a large population-based cohort with serological data, higher intakes of fruits and vegetables and dietary fiber were consistently associated with a lower probability of SARS-CoV-2 infection [1]. A systematic review of observational studies similarly concluded that higher diet quality and greater consumption of fruits, vegetables, and fiber tend to be associated with reduced risk of SARS-CoV-2 infection and/or COVID-19 severity, although causality cannot be inferred from these study designs [2]. In patients hospitalized with COVID-19, higher reported intakes of fruits, vegetables, and dietary fiber were inversely associated with disease severity and symptom burden in cross-sectional analyses [3]. Collectively, these findings support the view that diet may be a modifiable factor related to respiratory infection susceptibility and subsequent clinical course.

Several biological pathways provide plausibility for diet–infection associations. Nutrient-dense, minimally processed dietary patterns support epithelial barrier integrity, immune competence, and regulated inflammatory responses [4]. Dietary fiber is of particular interest because it shapes gut microbiota composition and metabolism and promotes the production of short-chain fatty acids (SCFAs), which can influence mucosal immunity and systemic inflammatory tone [5,6]. Experimental work also supports the concept of a gut–lung immune axis, whereby microbial metabolism of fermentable fiber can affect immune responses in the respiratory tract [7]. Beyond fiber, multiple micronutrients may contribute directly to immune regulation [8]. Among several micronutrients implicated in immune regulation, magnesium has recently received increased attention. Magnesium homeostasis has been discussed as relevant in COVID-19, consistent with magnesium’s roles in immune cell function and inflammatory control [9]. Mechanistically, extracellular magnesium can act as a signal, shaping T-cell effector function via LFA-1-dependent pathways [10]. Importantly, higher intake of fiber and magnesium may, therefore, represent both specific immunoregulatory influences and practical indicators of an overall nutrient-dense dietary pattern and the immune-supportive and anti-inflammatory properties of the diet.

Our group has contributed to this field by investigating diet-related predictors of COVID-19 risk in young adults without comorbidities. We previously reported that favorable nutritional behaviors—particularly consuming more than 500 g/day of vegetables and fruit and more than 10 g/day of nuts—and characteristic gut microbiota features were associated with a lower risk of COVID-19 in this population [11]. A reduction in infection is the single strongest indicator of a better functioning immune system: in discussing biomarkers of immunity in humans, Albers et al. concluded “no single marker allows conclusions to be drawn about the modulation of the whole immune system, except for the clinical outcome of infection itself”, indicating that infection is the most robust marker of the ability to mount an effective immune response [12]. Building on these observations, we developed the POLA index to quantify the immunomodulatory potential of the diet and demonstrated that a beneficial POLA index was associated with a lower COVID-19 incidence and with characteristic gut microbiota features [13]. Importantly, we also showed a very strong correlation between the POLA index and the dietary inflammatory index (DII) (r = 0.90; *p* < 0.0001). The POLA index can, therefore, also classify an individual’s diet on a scale ranging from strongly anti-inflammatory (0 points) to strongly pro-inflammatory (19 points). [13]. In a subsequent pilot cohort study in young women, we provided further support for the POLA index concept in relation to COVID-19 and influenza incidence, and selected markers of mucosal immunity [14]. These studies, while supportive, were limited by modest sample sizes and underline the need for methodological simplification and external validation.

While comprehensive indices, such as POLA, are valuable for detailed dietary evaluation and for identifying specific areas for nutritional improvement, their multi-component structure limits feasibility for rapid screening and routine counseling. A key translational question is, therefore, whether a small set of simple dietary indicators can capture a similar signal to that provided by a complex dietary index. Conceptually, the progress of the POLA index can be viewed as a necessary step towards identifying a simpler screening metric that captures a substantial portion of the signal provided by the comprehensive index (i.e., POLA) but with minimal burden.

Interpretable data-driven methods may help to identify such minimal, actionable indicators. Interactive tree approaches are widely used in medical and nutritional research because they can accommodate non-linear relationships and interactions while yielding transparent “if–then” rules and clinically intuitive cut-offs [15]. Accordingly, the aim of this study was to identify prognostic factors using interactive trees that would yield results similar to those of the overall POLA index, taking into account the lower risk of contracting COVID-19 or influenza among young people without comorbidities. We hypothesized that dietary fiber intake, together with magnesium adequacy, would emerge as key discriminators and would approximate POLA-based classification, with the full POLA index remaining useful for comprehensive assessment and individualized optimization.

## 2. Materials and Methods

### 2.1. Study Design and Participants

Two groups of participants were included in this study. The first group consisted of 95 people (73 men and 22 women), with a mean age of 34.26 ± 5.76 years and a mean body mass index (BMI) of 23.32 ± 2.76 kg/m^2^, who participated in this study in 2020. A detailed description of the group is presented elsewhere [11]. The second group consisted of 51 women, with an average age of 35.0 ± 5.6 years and an average BMI of 22.4 ± 2.3 kg/m^2^, who participated in this study in 2022. A detailed description of the group is presented elsewhere [14]. Due to differences in the recruitment period and cohort composition, the recruitment phase was taken into account in all multivariate analyses.

This prospective observational study was carried out in two waves (2020: first cohort; 2022: second cohort) at the Department of Nutrition and Drug Research, Jagiellonian University Medical College (Kraków, Poland), within an institutional research program. Eligible participants were healthy adults aged 25–45 years (no comorbidities) who had adhered to either a vegetarian diet or a traditional omnivorous diet for at least 12 months. Recruitment was conducted in Kraków using social media postings and participant referral (snowball sampling). Individuals who expressed interest contacted the research team and were screened for eligibility. To capture habitual lifestyle behaviors, data collection was scheduled for a 7-day observation period; if an atypical event (e.g., wedding or birthday) was planned, the observation was postponed to the following week. During this week, participants were asked to maintain their usual dietary and physical activity habits. Inclusion criteria were as follows: age 25–45 years; absence of chronic disease; BMI 18.5–29.9 kg/m^2^; stable vegetarian or omnivorous dietary pattern for ≥12 months; and no use of antibiotics or probiotics within the preceding 3 months. In total, 146 participants were enrolled (traditional diet: *n* = 90; vegetarian diet: *n* = 56). Written informed consent was obtained from all participants prior to participation. This study was conducted in accordance with the Declaration of Helsinki for medical research and with the positive approval of the Jagiellonian University Bioethics Commission (No. 1072.6120.5.2020 and 1072.6120.202.2019).

### 2.2. Anthropometry and Body Composition

To reduce potential measurement variability, the participants were asked to refrain from strenuous exercise for 24 h before assessment and to void their bladder (if needed). Height was recorded under standardized conditions (barefoot and standing upright) to the nearest 0.1 cm using a portable stadiometer (Seca 213, Seca GmbH & Co., Hamburg, Germany). Body mass and segmental body composition were measured with a calibrated bioelectrical impedance analyzer (Tanita BC-418 MA, Tanita, Tokyo, Japan), operating at 6.25 and 50 kHz (90 µA). Body weight was recorded to the nearest 0.1 kg, and body composition parameters to the nearest 0.1%. Waist circumference was measured using a non-elastic measuring tape (Seca GmbH & Co., Hamburg, Germany) to the nearest 0.1 cm under standardized conditions (standing position and abdomen relaxed), at the midpoint between the lower margin of the last palpable rib and the top of the iliac crest at the end of a normal expiration. The waist-to-height ratio (WHtR) was calculated as the waist circumference (cm) divided by height (cm). BMI was calculated as weight in kilograms divided by height in meters squared (kg/m^2^).

### 2.3. Physical Activity Monitoring

Habitual physical activity and sleep were assessed over seven consecutive days using a Polar M430 (Polar Electro Oy, Kempele, Finland) wrist-worn monitor placed on the non-dominant wrist. The participants were asked to wear the device continuously (24 h/day) and to remove it only for bathing. The monitor provided measures of total energy expenditure, daily step count, and sleep duration. Each participant received standardized instructions for device use in both written form and as a verbal briefing.

### 2.4. Dietary Assessment

Before the observation week, participants were trained on how to complete a 7-day prospective food diary and were provided with practical examples. To improve portion-size accuracy, they were encouraged to weigh foods using kitchen scales or, when weighing was not feasible, refer to a website with photos of products and their weight (http://www.ilewazy.pl, accessed on 21 March 2026). The participants recorded all foods and beverages consumed, as well as vitamins and minerals in the form of supplements, throughout the 7-day period. At the end of the week, diaries were reviewed together with each participant to check for missing entries and to clarify uncertain items. The dietary data were then entered into the “Dieta 6.0” software (National Food and Nutrition Institute, Warsaw, Poland) to estimate daily energy intake and nutrient intake, including macronutrients, dietary fiber, cholesterol, vitamins, and minerals. Nutrient adequacy was evaluated using Polish age- and sex-specific dietary reference values (RDA, AI, or EAR, as applicable) [16].

### 2.5. POLA Index Calculation

Detailed information regarding the theoretical assumptions and the calculation of the POLA index is presented elsewhere [14].

The dietary data collected during the 7-day monitoring period were used to compute the POLA index for each participant. To ensure adequate dietary coverage, only individuals with at least five completed diary days, including a minimum of three weekdays and two weekend days, were eligible for POLA calculation. For each participant, the mean daily intake was calculated across all available recording days.

The POLA index comprises the adequacy of selected nutrients and food-based components considered relevant to immune function and overall diet quality. Specifically, the following components were included: potassium, magnesium, iron, zinc, calcium, vitamin A, vitamin E, thiamin, vitamin B6, vitamin C, vitamin D, linoleic acid (LA), α-linolenic acid (ALA), folates, and dietary fiber, as well as consumption of fruits and vegetables (combined) and nuts. For most components, intake adequacy was evaluated against age- and sex-specific Polish reference values (RDA, AI, or EAR, as applicable). The participants received 0 points when their mean intake reached at least 100% of the relevant reference value, and 1 point when intake was below 100%.

Two components were scored using a three-level approach to better reflect clinically meaningful thresholds. First, vitamin D intake was categorized as ≥100%, 50–<100%, or <50% of the reference value (15 µg/day), and scored 0, 1, or 2 points, respectively. Second, combined fruit and vegetable intake was categorized as ≥600 g/day, 400–<600 g/day, or <400 g/day, and scored 0, 1, or 2 points, respectively. This food-based scoring was intended to capture the contribution of whole-food bioactives that may not be fully represented in nutrient databases. In addition, nut consumption was scored dichotomously: ≥10 g/day was assigned 0 points, and <10 g/day was assigned 1 point.

The total POLA score was calculated by summing points across all components, with higher scores indicating a less favorable dietary profile. Scores were classified into three categories: ≤5 points, beneficial immunomodulation (BIM); 6–11 points, unbeneficial immunomodulation (UBIM); and ≥12 points, highly unbeneficial immunomodulation (HUBIM). For statistical analyses, UBIM and HUBIM were combined into a single category (UBIM + HUBIM).

### 2.6. Statistical Analysis

Continuous variables are presented as the mean ± standard deviation (SD) for approximately normally distributed data, or as median with an interquartile range (Q1–Q3) otherwise. Normality was assessed using the Shapiro–Wilk test. Between-group comparisons were performed using the independent-samples Student’s *t*-test or the Mann–Whitney U test, as appropriate. Categorical variables were compared using the Chi-square test or Fisher’s exact test. Associations between quantitative variables were evaluated with Spearman’s rank correlation coefficient. Multivariable logistic regression was used to estimate odds ratios (ORs) with 95% confidence intervals (CIs) for the association between dietary screening categories (e.g., RapidPOLA or StrongPOLA) and the incidence of COVID-19 or influenza during follow-up. The adjusted models included the following covariates, selected a priori: sex, type of diet (vegetarian vs. omnivorous), physical activity level, marital status, year of cohort (2020 vs. 2022) and WHtR. Complete-case analysis was applied.

All analyses were conducted using PS IMAGO PRO 10 (IBM SPSS Statistics 29, IBM Corp., Armonk, NY, USA) and STATISTICA 13.3 (TIBCO Software Inc., Palo Alto, CA, USA). A two-sided *p*-value < 0.05 was considered statistically significant.

### 2.7. Data Mining

To identify a minimal and interpretable set of baseline dietary and lifestyle features associated with subsequent infection, we applied classification and regression tree (CART; C&RT) analysis, using interactive tree Analysis in STATISTICA 13.3 software. The candidate predictors included sociodemographic variables, diet type, and selected nutrient adequacy measures derived from the 7-day dietary records. The outcome was the incidence of COVID-19 or influenza during follow-up (yes/no).

The tree was grown using standard impurity-based splitting (Gini index) with pre-defined stopping rules (minimum tree depth was one, maximum tree depth was two), whereas the stopping rule was to prune in case of misclassification. The final tree was selected based on misclassification cost/overall classification performance and interpretability. Cut-off points identified by the interactive trees were subsequently used to define simplified screening categories (RapidPOLA and StrongPOLA) evaluated in regression analyses. These classifications and cut-offs were internally derived from the present dataset and should not be interpreted as externally validated clinical thresholds. Furthermore, because no external validation dataset was available, all analyses involving RapidPOLA and StrongPOLA should be considered hypothesis-generating.

### 2.8. Follow-Up Outcome Ascertainment

Follow-up was conducted separately for each recruitment wave. In June 2021, the participants from the 2020 cohort were contacted and asked whether they had developed COVID-19 after baseline assessment. COVID-19 was considered confirmed if the participants reported a positive polymerase chain reaction (PCR) test and/or a positive SARS-CoV-2 antibody test.

In June 2023, the participants from the 2022 cohort were contacted to determine whether they had developed COVID-19 or influenza since baseline. In this cohort, outcomes were self-reported, and no laboratory confirmation or formal case definition was applied. This was due to the fact that very few people had been getting tested for COVID-19 or influenza since the beginning of 2022. Participation was voluntary, and all questions were optional; the response rate was 100% for both the 2020 and 2022 cohorts. Reported infections were analyzed in relation to baseline dietary characteristics (including POLA category and simplified screening indicators) and physical activity measures. Given the differences between cohorts in terms of testing for COVID-19 or influenza, the misclassification of results is to be expected; therefore, this was accounted for in the multivariate analyses.

## 3. Results

### 3.1. Characteristics of Participants

This study included 146 young adults, 73 men (50.0%) and 73 women (50.0%). The mean age of the participants was 34.9 ± 5.6 years. The descriptive characteristics of the study group are presented in Table 1. A total of 39 participants (26.7%) reported having experienced either COVID-19 or influenza during the follow-up period. None of the participants required hospitalization. The POLA index was higher in those who reported COVID-19 or influenza infection than in those who did not (10.7 ± 4.4 vs. 7.8 ± 4.5; *p* = 0.0008).

### 3.2. Data Mining—The Use of Interactive Trees

In order to identify the factors most strongly associated with the risk of contracting COVID-19 or influenza, the following variables were introduced into the interactive tree model.

Qualitative variables: type of diet, sex, education, marital status, and BMI category.

Quantitative variables: age, PAL, % of the recommended intake for energy, water, protein, potassium, calcium, magnesium, iron, zinc, vitamin A, vitamin E, thiamine, riboflavin, niacin, vitamin B6, vitamin C, LA, ALA, folates, vitamin B12, and vitamin D, and a new indicator: fiber in g per kg/m^2^ of BMI.

**Figure 1:**
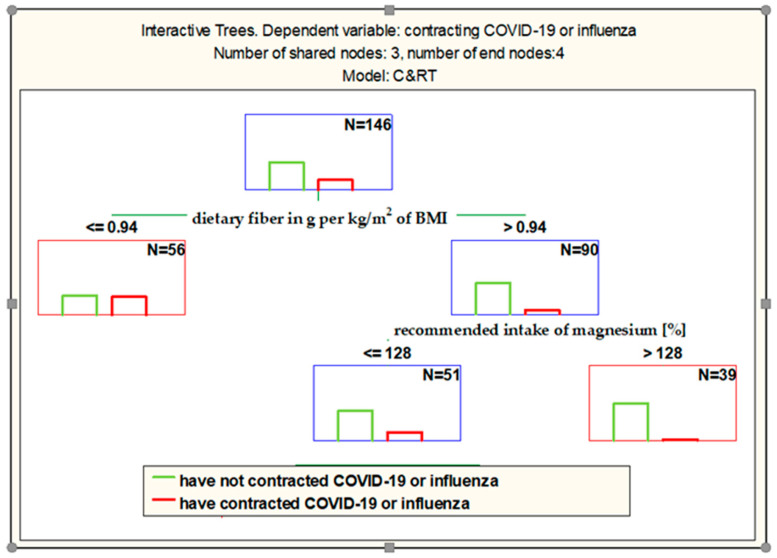
Result of interactive trees modeling.

The C&RT model was used, with the maximum number of levels set to 2 in the stopping parameters. The factors that proved to be most strongly associated with a reduced risk of COVID-19 or influenza were a diet in which the fiber intake in g per kg/m^2^ of BMI was at least 0.94 g/(kg/m^2^) and magnesium intake was at least 128% of the recommended value (i.e., 410 mg/day for women and 538 mg/day for men) (Figure 1).

**Figure 2:**
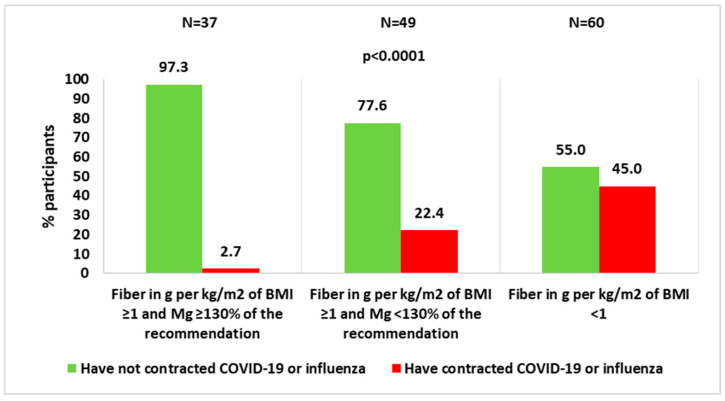
Incidence of COVID-19 or influenza during follow-up by dietary fiber and magnesium intake category.

Based on the results obtained, the following decisions were made:

- For ease of reference, a cutoff point of 1 g fiber per kg/m^2^ of BMI and 130% of the recommended intake for magnesium (i.e., 416 mg/day for women and 546 mg/day for men) was adopted.
- A division into three groups was adopted: group 1 with fiber in g per kg/m^2^ of BMI ≥ 1 and magnesium ≥ 130% of the recommendation, group 2 with fiber in g per kg/m^2^ of BMI ≥ 1 and magnesium < 130% of the recommendation, and group 3 with fiber in g per kg/m^2^ of BMI < 1 (Figure 2).

The participants who consumed <1 g fiber per kg/m^2^ of BMI had higher adjusted odds of reporting COVID-19 or influenza during follow-up compared with those who consumed ≥1 g fiber per kg/m^2^ of BMI and ≥130% of the magnesium reference value. However, the effect estimate was large and imprecise, with a wide confidence interval (adjusted OR = 23.1, 95% CI: 2.68–198.96), indicating that this result should be interpreted cautiously (Table 2). Therefore, dietary intake of fiber and magnesium was used to define the StrongPOLA index, with those consuming ≥1 g fiber per kg/m^2^ of BMI and ≥130% of the recommended amount of magnesium having the lowest risk of respiratory tract infections. The only statistically significant variable in the model was the StrongPOLA index; the other variables were not statistically significant (Table 2).

The detailed results (sociodemographics and % of vitamin and mineral requirements met, as well as consumption of vegetables, fruit, etc.) according to the StrongPOLA index are presented in the Supplementary Materials (Tables S1–S4).

Next, considering how strongly dietary fiber in g per kg/m^2^ of BMI and magnesium intake were associated with COVID-19 or influenza cases, we checked whether these two parameters yield similar results to the overall POLA index. The participants were divided into two groups: the first group consisted of individuals whose fiber intake in g per kg/m^2^ of BMI ≥ 1 and whose magnesium intake was at least 110% (this was decided arbitrarily) of the recommended daily allowance (i.e., 352 mg/day for women and 462 mg/day for men), while the second group consisted of the remaining participants. This dichotomy was used to define the RapidPOLA index. Given that the diet of the first group was NUTRIent-Dense, IMmune-supportive, and Anti-inFlammatory (NUTRIDIMAF), we introduced the acronym “NUTRIDIMAF diet” and also “strongNUTRIDIMAF diet” for a favorable StrongPOLA profile.

### 3.3. Characteristics of Participants According to RapidPOLA Groups

There were no significant differences in age, height, body weight and physical activity level (PAL) between participants according to the RapidPOLA index. However, a difference was observed in BMI and WHtR indices. The participants with a higher dietary intake of fiber and magnesium had lower values for these two indices. The distribution of participant sex, smoking and education did not differ according to the RapidPOLA index. However, differences in the type of diet and marital status were found according to the RapidPOLA index: among the participants with high intake of dietary fiber and magnesium, there were significantly more vegetarians and single/divorced participants than among others. In addition, differences were noted in the body fat categories, WHtR, physical activity and use of vitamin supplements between groups. Among participants with higher fiber and magnesium intake, there were fewer people with excess body fat, while more were physically active in their free time and more used vitamin supplements (Table 3).

In the study group, 58 participants consumed a diet with a high level of dietary fiber and magnesium, and the remaining 88 participants were on a diet where intake of dietary fiber was below 1 g per kg/m^2^ of BMI or magnesium was <110% of the recommended intake. The median fiber intake was 33.1 and 19.5 g/day in the two groups, while the median magnesium intake was 529.5 and 339 mg/day. A significant difference was observed between the groups in the POLA index (4.5 ± 2.6 vs. 11.2 ± 3.7), indicating a strong immune-supportive and anti-inflammatory effect of the NUTRIDIMAF diet. There were significant differences in the intake of many other dietary constituents between these groups (i.e., according to the RapidPOLA index). Intake of energy, water, protein, plant protein, fat, LA, ALA, omega-6 fatty acids, carbohydrates, and all vitamins (except niacin) and minerals was higher in the group with higher fiber and magnesium intake (Table 4). However, there were no differences in the intake of alcohol and vitamin D between the two groups (Table 4).

In the group with high intake of fiber and magnesium, dietary intake recommendations were achieved for at least 75% of the participants (looking at the first quartile—Q1), apart from those for vitamin D, LA, ALA, iron, and calcium (Table 5). On the other hand, at least 75% of the participants in the “other” group did not meet the intake recommendations for LA, fiber, calcium, folates, and vitamin D (Table 5).

There were significant differences in the intake of selected foods according to the RapidPOLA index, as presented in Table 6. The participants in the group with high dietary fiber and magnesium showed a significantly higher consumption of groats and rice, seeds, nuts, fruits, vegetables, and legumes than the participants in the “other” group. Similar differences between the groups were noted for vegetables, fruits, and nuts after these products were divided according to average intake (more than 600 g for vegetables and fruits, and more than 10 g for nuts).

### 3.4. POLA Index or RapidPOLA Index and Risk of COVID-19 or Influenza

**Figure 3:**
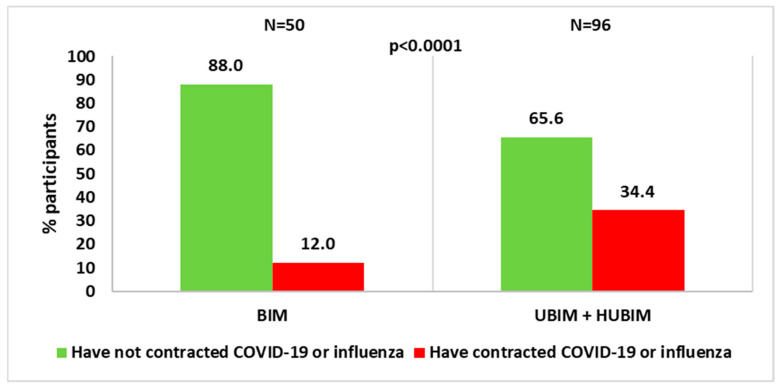
Incidence of COVID-19 or influenza during follow-up according to POLA index categories (BIM vs. UBIM + HUBIM).

The percentage of the participants who reported COVID-19 or influenza during the follow-up period, compared to those who did not, stratified by the POLA index category, is shown in Figure 3. In the BIM group, only 12% reported infection after baseline, compared to 34.4% in the UBIM + HUBIM group (*p* < 0.0001).

**Figure 4:**
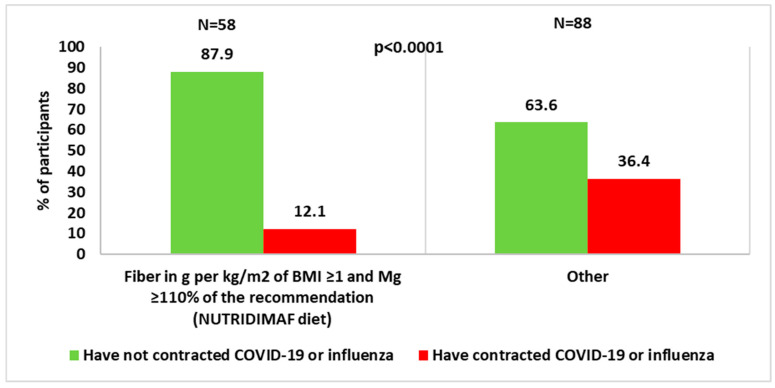
Incidence of COVID-19 or influenza during follow-up according to RapidPOLA index.

The percentage of the participants who reported COVID-19 or influenza during the follow-up period, compared to those who did not, stratified by RapidPOLA index category, is shown in Figure 4. In the group with a high level of dietary fiber and magnesium, only 12.1% reported infection after baseline, compared to 36.4% in the other group (*p* < 0.0001).

The odds of reporting COVID-19 or influenza according to POLA and RapidPOLA categories are shown in Table 7. Participants in the UBIM + HUBIM group had higher odds of illness compared to those in the BIM group. In the unadjusted model (Model 1), the OR for UBIM + HUBIM vs. BIM was 3.8 (95% CI: 1.48–9.45). This association remained elevated in the fully adjusted model controlling for potential confounders (Model 2: OR = 3.0; 95% CI: 1.18–8.07; *p* < 0.05). Based on the RapidPOLA index, the group with a high level of dietary fiber and magnesium showed about 76% lower odds of COVID-19 or influenza than the other participants. The result remained significant regardless of the level of adjustment (OR = 3.4, 95%CI: 1.28–8.75 in the fully adjusted model).

### 3.5. Correlations Between POLA Index and Dietary Fiber and Magnesium Intake

**Figure 5:**
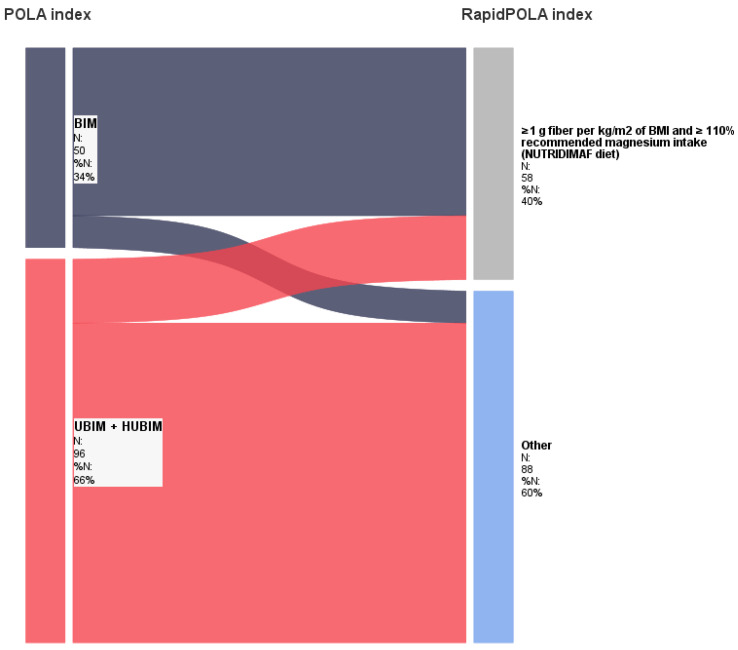
A Sankey diagram showing the agreement of the POLA and RapidPOLA indexes.

There was a strong correlation between the POLA index and dietary fiber in g per kg/m^2^ of BMI and magnesium % recommended (r = −0.80; *p* < 0.0001 and r = −0.79; *p* < 0.0001, respectively) as presented in Table 8. Moreover, fiber and magnesium were strongly correlated with most components of the POLA index. Cohen’s kappa test showed good agreement in the qualitative interpretation between the BIM vs. UBIM + HUBIM in the POLA index and RapidPOLA index (kappa = 0.65; *p* < 0.0001) (Figure 5).

### 3.6. Comparison of Eating Habits Between People Who Eat Traditionally and Vegetarians for Group with ≥1 g Fiber per kg/m^2^ of BMI and ≥110% Magnesium Intake Recommendation

**Figure 6:**
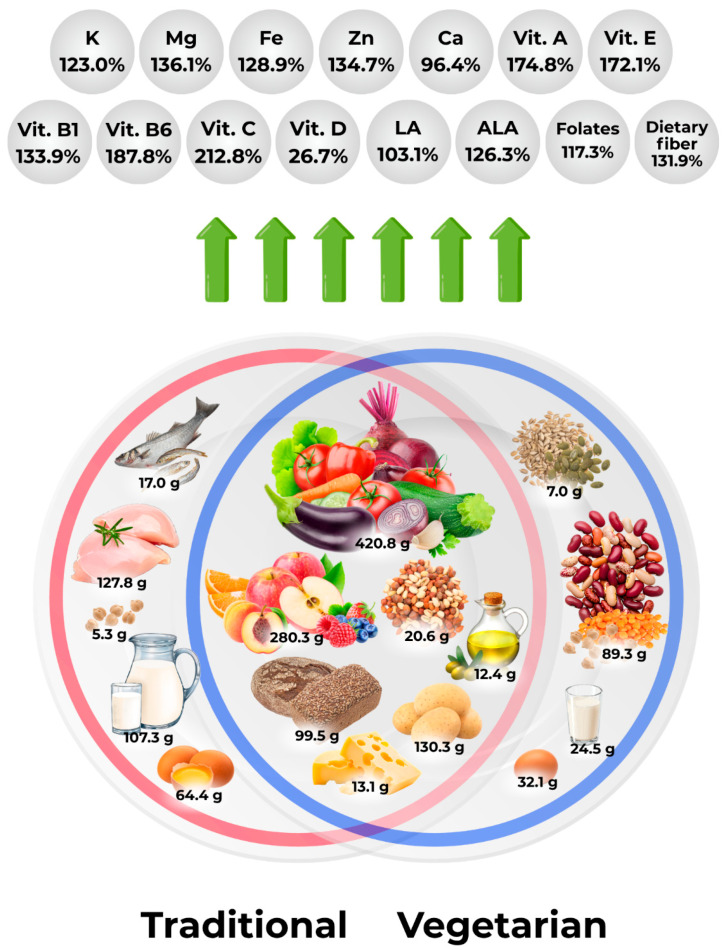
A graphical summary of the consumption of selected foods according to the type of diet and the percentage of the recommended intake for the group with ≥1 g fiber per kg/m^2^ of BMI and ≥110% magnesium recommended intake (NUTRIDIMAF diet) (plate-based guidance).

No differences were found in the energy content of the diet, or in the supply of total fats, total carbohydrates, and most vitamins and minerals included in the POLA index, or in the POLA index score between the type of diet for those with >92% dietary fiber and >110% magnesium (Table 9). When analyzing the consumption of selected food types between these groups, it was noted that the participants following a traditional diet obviously ate more meat, eggs, fish, and milk than vegetarians, while the vegetarians consumed more legumes, seeds, groats, and rice. Importantly, there were no differences in the consumption of nuts, fruits, and vegetables; the consumption of these food groups was a “common denominator” guaranteeing the supply of vitamins, minerals, fiber, and essential fatty acids (Table 10). A graphical summary of selected information from Table 5 and Table 10, referring to the Healthy Eating Plate, is presented in Figure 6.

## 4. Discussion

This prospective observational study shows that, in a cohort of adults aged 25–45 years who are not obese, without comorbidities, are non-smokers, and engage in moderate physical activity, better overall diet quality was associated with a lower follow-up incidence of COVID-19 or influenza. Across the full POLA index and the simplified interactive tree-derived rules (StrongPOLA and RapidPOLA), the most informative markers were dietary fiber in g per kg/m^2^ of BMI and magnesium adequacy. Importantly, these two variables should be interpreted primarily as practical indicators of a broader nutrient-dense dietary pattern rather than as isolated causal factors.

The strongest separation of risk was observed according to the StrongPOLA profile, although the large effect estimates should be interpreted cautiously, given wide confidence intervals and strongly contrasting lifestyle profiles. Importantly, however, in multivariable logistic regression, the only statistically significant variable in the model was the StrongPOLA index; the other variables (diet, sex, marital status, physical activity, WHtR, and cohort year) were not statistically significant. The participants consuming less than 1 g fiber per kg/m^2^ of BMI had substantially higher adjusted odds of respiratory illness than those meeting both the fiber and higher magnesium criteria (≥1 g fiber intake per kg/m^2^ of BMI and magnesium intake at ≥130% of the recommendation). This suggests that inadequate fiber intake is a particularly useful warning sign of a less favorable overall dietary pattern in this cohort.

This interpretation is consistent with the biological and epidemiological literature. Fiber-rich diets are typically characterized by higher intakes of vegetables, fruits, legumes, nuts, whole grains, vitamins, minerals, essential fatty acids, and phytochemicals, and fiber itself supports the gut microbiota and short-chain fatty acid production, both of which are relevant to immune regulation [3,17,18,19,20,21,22,23]. Furthermore, recent findings from a study using data from 103,649 participants in the UK Biobank indicate that a high fiber intake has the greatest beneficial impact on mortality rates [24].

Magnesium likely complements this pattern. In our study, the RapidPOLA-positive group had substantially higher magnesium intake, but also a more favorable overall nutrient profile. Thus, magnesium appears to improve discrimination of the healthiest dietary profile, while fiber remains the clearest marker of diet quality at the screening level [21,25,26,27,28,29].

In this context, the lower observed incidence of respiratory illness among the participants with higher dietary fiber and magnesium intake should be interpreted not as a direct protective effect of these two dietary constituents, but as an association with an overall healthier dietary pattern that is biologically plausible to support immune function and inflammatory balance.

### 4.1. The Components of the POLA Index and the Immune System

The POLA index takes into account the supply of minerals, vitamins, and fatty acids that have a proven immune-supportive effect, including potassium, magnesium, iron, zinc, calcium, vitamins A, D, E, B1, B6, B9 (folic acid) and C, linoleic acid (18:2*n*-6), and α-linolenic acid (C18:3*n*-3), as well as intake of fiber, fruits, vegetables, and nuts [13]. Fruits, vegetables, and nuts are good sources of fiber, many of the nutrients included in the POLA index, and polyphenols. Fiber is important in supporting a healthy, diverse gut microbiota, which in turn shapes and supports the immune response and helps to modulate inflammation [30,31,32,33]. Many of the effects of fiber are mediated by SCFAs, which act to maintain the gut epithelial barrier but also interact with the gut-associated immune system, although other mechanisms are also involved [34]. Many of the vitamins and minerals support the metabolic machinery of immune cells that are involved in energy generation, in biosynthesis, for example, of proteins involved in the response (cytokines, acute-phase proteins, and antibodies), and in cellular proliferation [35]. Consequently, those nutrients are vital in supporting an effective immune response, including promoting barrier function, cellular aspects of innate immunity, and the function of antigen-presenting T- and B-cells [8,36,37,38,39], while zinc has multiple direct anti-viral actions, for example, inhibiting the replication of RNA viruses through the inhibition of viral RNA polymerases [40]. With regard to barrier function, vitamin A promotes epithelial differentiation and integrity, gut homing of T- and B-cells, and secretory IgA production; vitamin B6 promotes gut homing of T-cells; folic acid is a survival factor of regulatory T-cells in the gut-associated immune system; vitamin D promotes epithelial tight junction integrity via the production of E-cadherin and connexin 43; and zinc and iron maintain the integrity of the epithelial barrier [8,36,37]. Within the innate immune system, vitamins A, B6, C, and E, folic acid, zinc, and iron all support the activity of natural killer cells, which are directly involved in anti-viral immunity, while vitamins A, C, and D, zinc, and iron support phagocytic activity and microbial killing [8,36,37]. Vitamin A regulates the development and differentiation of different helper T-cell phenotypes, promotes the development of regulatory T-cells, regulates the production of key immunoregulatory cytokines by T-cells, and supports the function of B-cells and antibody production [41,42]. Vitamin B6, folic acid, and vitamin E also regulate the development and differentiation of different helper T-cell phenotypes, promote the function of type-1 helper T-cells involved in antibacterial and anti-viral defense, and support the function of B-cells and antibody production [43,44]. Vitamin C regulates the development and differentiation of different helper T-cell phenotypes, promoting the generation of cytotoxic T-cells involved in anti-viral defense, and supports the function of B-cells and antibody production [45]. Vitamin D regulates the development and differentiation of different helper T-cell phenotypes, promoting the development of regulatory T-cells [46,47,48,49,50]. Zinc regulates the development and differentiation of different helper T-cell phenotypes, promoting the development of both cytotoxic and regulatory T-cells, promoting the production of key immunoregulatory cytokines by T-cells (interleukin-2), and interferon-γand supports the function of B-cells and antibody production [51,52]. Magnesium promotes macrophage, helper T-cell, and B-cell function, including anti-viral responses and antibody production by B-cells [26]. The essential fatty acids (linoleic and α-linolenic) are required for the synthesis of phospholipids involved in cell membrane structure, supporting the proliferation of immune cells, and are the substrates for the synthesis of longer-chain, more unsaturated polyunsaturated fatty acids also required for cell membrane phospholipids and as precursors for immunoregulatory lipid mediators [53,54,55]. It is immediately evident that insufficient intake of one or more of these essential nutrients (vitamins, minerals, and essential fatty acids) will impair the immune response, making the host more susceptible to infection. On the other hand, sufficient intake of all of these essential nutrients will provide optimal support to the immune system, including barrier function, innate immunity, and T- and B-cell function, lowering the risk for becoming infected and for infections becoming more severe. This explains the strength of the POLA index in predicting risk and severity of viral infection. However, in the current study, we identify that the simpler StrongPOLA and RapidPOLA indexes are simple metrics that reflect the quality of a diet without the need to analyze all of its components in detail. They serve as an indicator of nutritional density and the immune-supportive and anti-inflammatory potential of the diet; these each require one and the same thing: a high-quality diet.

### 4.2. POLA Index, RapidPOLA, StrongPOLA, and Incidence of COVID-19 or Influenza

In this internally derived analysis, RapidPOLA showed promising agreement with the full POLA classification. The participants meeting the cut-offs of ≥1 g fiber intake per kg/m^2^ of BMI and ≥110% of recommended magnesium intake (NUTRIDIMAF diet) had a markedly lower incidence of respiratory illness during follow-up, and RapidPOLA showed strong correlation and good agreement with the full POLA classification. Nevertheless, RapidPOLA should be considered a candidate screening classification that requires external validation before it can be recommended for clinical or public-health use.

In our study, the participants consuming the NUTRIDIMAF diet consumed 530 mg of magnesium/day (median) and 33.1 g dietary fiber/day (median), while in the remaining group, the intakes were only 339 mg/day (median) and 19.5 g/day, respectively. Although RapidPOLA is based on only two nutritional parameters, the participants meeting its criteria consumed more vegetables and fruit (median 680 g/day), nuts (20.6 g/day), seeds, legumes, hard cheese, groats and rice, meat, and fish, which resulted in a greater intake of protein (especially plant protein), unsaturated fatty acids, fiber, and most vitamins and minerals. In addition, this group reported higher dietary water intake, a component essential for the proper functioning of dietary fiber and beneficial gut microbiota. Therefore, RapidPOLA appears to capture a broad food-based pattern rather than relating to a narrow effect of a few nutrients. Importantly, the POLA index shows a very strong correlation with the DII (r = 0.90); a POLA score of 4.5 on average—as indicated by the preferred RapidPOLA result—signifies that such a diet has a strongly anti-inflammatory profile, as well as supporting the immune system to function.

From a practical perspective, RapidPOLA may be useful as a first-line screen: when both fiber and magnesium thresholds are met, the overall dietary profile is likely to resemble the favorable POLA pattern (BIM); when they are not met, a more detailed POLA-based assessment may help identify specific food groups and nutritional gaps. On this basis, we propose the use of the RapidPOLA index as a practical screening tool for assessing diet quality, corresponding to the POLA pattern. This fiber and magnesium intake-based profile is similar to a diet classified as BIM, suggesting that the supply of other nutrients important for immune system function is likely to be sufficient.

An important finding is that the favorable RapidPOLA profile was achievable in both dietary patterns examined, i.e., vegetarian and traditional. Within the RapidPOLA-positive group, traditional and vegetarian diets differed in their protein sources, but both shared a common core of foods—high intakes of vegetables, fruits, nuts, seeds, whole-grain products, and other minimally processed plant foods. This makes the result easy to translate into plate-based dietary guidance and suggests that any visual summary should emphasize shared core foods rather than diet labels alone. In this context, the results obtained are consistent with the Healthy Eating Plate concept recommended in Poland (National Centre for Nutrition Education) [56], which emphasizes the predominant role of fruit and vegetables in the daily diet, supplemented by whole grains and sources of protein, whilst limiting highly processed foods. This model reflects a dietary pattern with high nutrient density, similar to the profile identified by RapidPOLA as beneficial for the functioning of the immune system. It is worth noting that both the POLA and RapidPOLA indexes significantly differentiated the risk of disease. The participants classified as UBIM + HUBIM according to the POLA index had approximately a 3-fold higher risk of infection compared to the BIM group (OR = 3.0; 95% CI: 1.18–8.07), while RapidPOLA, based on only two nutritional parameters, showed a comparable ability to identify high- and low-risk groups.

The results obtained are also highly consistent with current U.S. dietary recommendations, which emphasize the consumption of unprocessed foods, a high intake of vegetables and nuts, adequate protein intake (1.2–1.6 g/kg body weight), and limiting saturated fatty acids to <10% of total energy intake [57].

The participants meeting the RapidPOLA criteria (NUTRIDIMAF diet) were also more physically active in their leisure time, which reinforces the broader lifestyle context of the findings. Diet quality, physical activity, adequate sleep, and absence of abdominal obesity probably cluster together and jointly contribute to resilience against respiratory infection.

StrongPOLA may be viewed as a stricter reference model. It identifies the subgroup with the most favorable observed combination of dietary characteristics and the lowest observed infection risk, whereas RapidPOLA provides a more practical and less restrictive screening approach. Both simplified indexes should, therefore, be interpreted as complementary to the full POLA index: RapidPOLA for rapid screening and counseling, and StrongPOLA as a more demanding benchmark profile.

The results obtained are particularly significant in the context of population data. Almost two decades ago, it was shown that a diet characterized by a fiber intake of at least 23 g/day and an adequate magnesium intake is associated with a lower severity of chronic inflammation, assessed, among other things, on the basis of hs-CRP levels [21]. While in 1996–2005 the average dietary fiber intake in Poland was 23.7 g/person/day, with significant variation (19.9–28.5 g/person/day) [58], national data indicate a clear decline in fiber intake in Poland, to around 17.8 g/day in 2017–2020 [59]. This means that a significant proportion of the population does not reach the levels observed in the groups with the lowest risk of infection in this study.

At the same time, this study is observational, and the very large odds ratios observed for StrongPOLA likely reflect strongly contrasting dietary and lifestyle profiles rather than the isolated effect of one or two nutrients. The results support further evaluation of simple dietary classifications, such as RapidPOLA and StrongPOLA, but external validation in independent cohorts is essential before broader implementation or use in clinical counseling, public-health screening, or risk stratification.

### 4.3. New Indicator “Dietary Fiber Intake in g per kg/m^2^” as Exploratory Guidance for Fiber Intake

Initially, the data analysis included dietary fiber as a percentage of the recommended daily intake (as in the POLA index) and magnesium as important dietary components for predicting respiratory tract infections. During further research, testing various options, it turned out that fiber intake relative to BMI and magnesium intake are even better predictive factors, and finally, dietary fiber intake in g per kg/m^2^ of BMI instead of dietary fiber in g was used in interactive tree analyses. This BMI-normalized fiber indicator aligns with the recommendation for personalized diets—guidelines tailored individually to each person. Furthermore, it is very easy to remember—at least 1 g of fiber per 1 kg/m^2^ of BMI, which will help healthcare professionals support people in meeting dietary recommendations. It is important to note that the 1 g per kg/m^2^ of BMI guidance is generally consistent with current recommendations for fiber intake. According to our findings, a person with a BMI of 25 kg/m^2^ should consume at least 25 g/day of fiber, based on lowering the risk of respiratory infection. In comparison, current recommendations for fiber intake by adults include 25 g/day by the European Food Safety Authority and Polish National Institute of Public Health, NIH—National Research Institute, 30 g/day by the UK Scientific Advisory Committee on Nutrition, and 28 g for every 2000 calories by the US authorities [16,60,61,62]. We recognize that this metric is derived solely from our current dataset; it is, therefore, exploratory and requires external validation.

### 4.4. Strengths and Limitations

The strengths of this study include the prospective design, the use of the same core methodology across two recruitment waves, the use of at least a 5-day dietary assessment, and the combination of conventional statistics with an interpretable interactive tree approach. A further strength is the evaluation of simplified screening rules against the full POLA index, which improves translational relevance. A major advantage of the StrongPOLA and RapidPOLA indexes is that they are “resistant” to errors resulting from the use of supplements. Fiber is very rarely supplemented, whereas foods that contain fiber (i.e., vegetables, fruits, grains, legumes, and nuts) also contain vitamins, minerals, essential fatty acids, protein, polyphenols, and fiber itself (for gut microbiota)—in other words, everything needed for the proper functioning of the immune system.

The main limitations are the observational design, the relatively small sample size, the inclusion of young and generally healthy adults only, and the reliance on self-reported dietary intake and follow-up outcomes in part of the cohort. Furthermore, no validity checks (Goldberg’s cut-off values) were applied in the analysis of the data from the food diaries. Our study results apply to individuals aged 25–45 years who are not obese, have no comorbidities, do not smoke, and engage in moderate physical activity. These features limit causal inference and the direct generalizability of the findings to older, obese, or chronically ill populations.

Accordingly, RapidPOLA and StrongPOLA should be regarded as promising hypothesis-generating and internally derived candidate screening classifications that warrant validation in larger and more diverse populations.

### 4.5. Implications and Future Directions

The present findings suggest that RapidPOLA could be evaluated in future studies as a quick food- and nutrient-based candidate screen for overall diet quality in the context of immune support (keeping in mind, however, that it is designed for young, non-obese individuals without comorbidities who engage in moderate physical activity). StrongPOLA may serve as a more ambitious, hypothetical reference profile associated with the lowest observed respiratory illness risk in this cohort. Future studies should test the cut-offs in external cohorts, in populations with different metabolic and clinical characteristics, and in intervention settings. As we wrote in our first paper about the POLA index, “Further studies should be carried out with a larger number of people, equally women and men, aged 18–65, both normal weight and obese, so that it can be applied to the general population. Such a study, in addition to an analysis of body composition, gut microbiota, energy expenditure, and eating behaviour, would also include blood sampling and thorough biochemical testing of parameters related to the immune system”. This is because individuals with obesity, smokers, and those with sedentary lifestyles, etc., in whom there may be impaired immunity and elevated chronic low-grade inflammation, most likely need to consume even more nutrient-rich foods to counteract the immune impairments and inflammation caused by obesity, physical inactivity, and smoking.

The use of data mining methods in the analysis of large nutritional and medical data opens up new possibilities for identifying hidden risk groups and complex relationships between diet, lifestyle, and health. At the same time, it should be emphasized that the results generated by data mining or Artificial Intelligence (AI) algorithms should always be interpreted in collaboration with experts, and only on this basis should practical and health recommendations be formulated.

## 5. Conclusions

In conclusion, this prospective observational study supports the usefulness of the POLA index for assessing diet quality in relation to immune-supportive dietary patterns, while also showing that two simple markers, dietary fiber and magnesium adequacy, appear to capture key elements of the signal provided by the full POLA index in a more practical way. The POLA index and the simpler StrongPOLA and RapidPOLA indexes have been tested on a group of people aged 25–45 years, who are non-obese, without comorbidities, non-smokers, and with moderate physical activity, and could currently be used for such people. However, further research is needed to confirm the effectiveness of these indicators for the more general population. On this basis, the following conclusions are drawn:

1. StrongPOLA, based on ≥1 g dietary fiber intake per kg/m^2^ of BMI and ≥130% of the magnesium reference value (i.e., 416 mg/day for women and 546 mg/day for men), identifies the subgroup with the lowest observed follow-up risk of COVID-19 or influenza in this cohort. However, because the associated odds ratios are large and imprecise, with wide confidence intervals, StrongPOLA should be treated as an exploratory reference profile rather than a validated risk-prediction tool.
2. RapidPOLA, based on ≥1 g fiber per kg/m^2^ of BMI and ≥110% of the magnesium reference value (i.e., 352 mg/day for women and 462 mg/day for men), shows good agreement with the favorable POLA profile (BIM). It could be considered a candidate screening classification for future validation studies. RapidPOLA index could serve as an indicator of a diet with high nutritional density, and with immune-supporting and anti-inflammatory potential (NUTRIDIMAF diet).
3. Fiber intake below 1 g per kg/m^2^ of BMI could be a practical warning signal of lower overall diet quality with pro-inflammatory effects and of a broader pattern of nutritional inadequacy in this cohort.
4. A recommendation to consume at least 1 g of fiber per 1 kg/m^2^ of BMI as an easy-to-remember guideline could be considered. Further research is needed before this can be adopted as a recommendation, as it would need to be supported by sufficient evidence and have been externally validated.
5. Both traditional and vegetarian diets could meet favorable immunonutritional criteria when they are built around vegetables, fruits, nuts, seeds, legumes, and other minimally processed nutrient-dense foods.
6. The use of data mining or AI methods in nutritional data analysis enables the identification of dietary patterns and risk groups that are less visible when using traditional approaches. However, such results should be interpreted together with nutritional, clinical, and public-health expertise before practical recommendations are formulated.
7. The results of our study contribute to the discussion regarding the need to raise the recommended intake levels for key nutrients that support immunity: potassium, magnesium, iron, zinc, calcium, vitamin A, vitamin E, thiamin, vitamin B6, vitamin C, vitamin D, LA, ALA, folates, and dietary fiber, especially during periods of increased risk of infection, such as the autumn and winter seasons. It is also worth considering whether vitamin and mineral intake should be recommended not only based on sex, age, and physical activity—as is currently the case—but also on individual BMI.
8. The findings are consistent with the importance of a holistic approach to health, including maintaining body weight, engaging in moderate physical activity, not smoking, getting adequate sleep, and having good eating habits, as essential elements for health maintenance.
9. We encourage researchers to test the utility of the RapidPOLA and StrongPOLA indexes both as markers of a good-quality diet and in identifying an increased risk of developing diseases for which inflammation is one of the etiological factors, such as obesity, cardiovascular disease, depression, cancer, autoimmune, and neurodegenerative diseases.
10. Because the current study was observational and conducted in healthy young adults, the proposed RapidPOLA and StrongPOLA cut-offs should be externally validated in independent and more diverse cohorts before clinical, public-health, or population-level application.

## Figures and Tables

**Table 1:** The anthropometric and sociodemographic characteristics of the study participants.

Variable		Total *n* = 146		Have Not Contracted COVID-19 or Influenza *n* = 107		Have Contracted COVID-19 or Influenza *n* = 39		Student’s *t*-Test *p*
		X ± SD		X ± SD		X ± SD		
Age [years]		34.9 ± 5.6		34.6 ± 5.6		35.6 ± 5.7		0.3553
Height [cm]		173.3 ± 9.3		174.1 ± 9.6		171.1 ± 8.4		0.0702
Body weight [kg]		69.5 ± 11.8		69.5 ± 12.1		69.2 ± 11.2		0.8804
BMI [kg/m^2^]		23 ± 2.6		22.8 ± 2.6		23.5 ± 2.5		0.1330
WHtR		0.47 ± 0.04		0.47 ± 0.04		0.48 ± 0.05		0.1798
TEE [kcal]		2420 ± 455		2433 ± 480		2384 ± 378		0.5182
PAL		1.5 ± 0.16		1.5 ± 0.17		1.5 ± 0.13		0.9631
Sleep duration [h]		7.26 ± 0.48		7.32 ± 0.48		7.12 ± 0.48		**0.0322**
Steps per day		12,600 ± 4433		12,754 ± 4708		12,180 ± 3590		0.4364
POLA index [points]		8.5 ± 4.6		7.8 ± 4.5		10.7 ± 4.4		**0.0008**
Variable	Category	*n*	%	*n*	%	*n*	%	Chi^2^ *p*
Sex	Men	73	50.0	55	51.4	18	46.2	0.5747
	Women	73	50.0	52	48.6	21	53.8	
Diet type	Traditional	90	61.6	61	57.0	29	74.4	0.0564
	Vegetarian	56	38.4	46	43.0	10	25.6	
BMI categories	Normal body weight	108	74.0	80	74.8	28	71.8	0.7173
	Overweight	38	26.0	27	25.2	11	28.2	
Marital status	Single/divorced	73	50.0	58	54.2	15	38.5	0.0923
	Married/cohabiting	73	50.0	49	45.8	24	61.5	
BF [%] categories	Underfat or Normal	119	82.1	89	84.0	30	76.9	0.3272
	Overfat	26	17.9	17	16.0	9	23.1	
Smoking status	No	128	87.7	95	88.8	33	84.6	0.4977
	Yes	18	12.3	12	11.2	6	15.4	
Education level	Secondary	9	6.2	8	7.5	1	2.6	0.2748
	Higher	137	93.8	99	92.5	38	97.4	

TEE, total energy expenditure; BMI, body mass index; BF, body fat; PAL, physical activity level; WHtR, waist-to-height ratio; *n*, number of participants; X, arithmetic mean; SD, standard deviation. The bold values denote statistical significance at the *p* < 0.05 level.

**Table 2:** The odds ratio for the relationship between the dietary fiber and magnesium intake category (StrongPOLA group) and developing COVID-19 or influenza.

Model		OR (95% CI)	OR (95% CI)
**StrongPOLA**	**Dietary Fiber in g per kg/m^2^** **of BMI ≥1 and Mg ≥ 130% Recommendation (strongNUTRIDIMAF diet)**	**Dietary Fiber in g per kg/m^2^** **of BMI ≥ 1 and Mg < 130%** **Recommendation**	**Dietary Fiber in g per kg/m^2^** **of BMI < 1**
Model 1 ^a^	1 (ref.)	10.4 (1.28–84.87)	29.5 (3.79–229.04)
Model 2 ^b^	1 (ref.)	11.4 (1.23–105.81)	23.1 (2.68–198.96)
**StrongPOLA**	**Dietary fiber in g per kg/m^2^** **of BMI ≥ 1 and Mg ≥ 130% recommendation (strongNUTRIDIMAF diet)**	**Other**	
Model 1 ^a^	1 (ref.)	19.3 (2.54–146.07)	
Model 2 ^b^	1 (ref.)	14.9 (1.89–118.06)	

^a^ crude model, ^b^ adjusted for type of diet, sex, marital status, physical activity, year of cohort and WHtR, OR—odds ratio.

**Table 3:** Characteristics of participants according to RapidPOLA groups.

Variable		Total *n* = 146		Dietary Fiber in g per kg/m^2^ of BMI ≥ 1 and Mg ≥ 110% Recommendation (NUTRIDIMAF Diet) *n* = 58		Others *n* = 88		Student’s *t*-Test *p*
		X ± SD		X ± SD		X ± SD		
Age [years]		34.9 ± 5.6		34.0 ± 5.6		35.5 ± 5.6		0.1195
Height [cm]		173.3 ± 9.3		173.6 ± 10.4		173.2 ± 8.6		0.8296
Body weight [kg]		69.5 ± 11.8		67.3 ± 12.8		70.9 ± 11.4		0.0890
BMI [kg/m^2^]		23.0 ± 2.6		22.2 ± 2.6		23.5 ± 2.6		**0.0025**
WHtR		0.47 ± 0.04		0.46 ± 0.04		0.48 ± 0.05		**0.0030**
TEE [kcal]		2420 ± 455		2401.8 ± 500.2		2432.7 ± 424.1		0.6998
PAL		1.5 ± 0.16		1.51 ± 0.19		1.49 ± 0.14		0.5755
Sleep duration [h]		7.26 ± 0.48		7.34 ± 0.47		7.21 ± 0.48		0.1063
Steps per day		12,600 ± 4433		13,103 ± 5091		12,269 ± 3935		0.2933
POLA index [points]		8.5 ± 4.6		4.5 ± 2.6		11.2 ± 3.7		**<0.0001**
Variable	Category	*n*	%	*n*	%	*n*	%	Chi^2^ *p*
Sex	Men	73	50.0	26	44.8	47	53.4	0.3102
	Women	73	50.0	32	55.2	41	46.6	
Diet type	Traditional	90	61.6	26	44.8	64	72.7	**0.0007**
	Vegetarian	56	38.4	32	55.2	24	27.3	
BMI categories	Normal body weight	108	74.0	49	84.5	59	67.0	**0.0188**
	Overweight	38	26.0	9	15.5	29	33.0	
Marital status	Single/divorced	73	50.0	37	63.8	36	40.9	**0.0068**
	Married/cohabiting	73	50.0	21	36.2	52	59.1	
BF [%] categories	Underfat or Normal	119	82.1	53	91.4	66	75.9	**0.0257**
	Overfat	26	17.9	5	8.6	21	24.1	
WHtR	<0.49	108	74.5	49	86.0	59	67.0	**0.0107**
	≥0.50	37	25.5	8	14.0	29	33.0	
Smoking status	No	128	87.7	54	93.1	74	84.1	0.1051
	Yes	18	12.3	4	6.9	14	15.9	
Education level	Secondary	9	6.2	4	6.9	5	5.7	0.7652
	Higher	137	93.8	54	93.1	83	94.3	
Self-rated physical activity (work)	Low	109	74.7	47	81.0	62	70.5	0.1223
	Moderate	32	21.9	11	19.0	21	23.9	
	High	5	3.4	0	0	5	5.7	
	Low	31	21.2	15	25.9	16	18.2	
Self-rated physical activity (leisure)	Moderate	78	53.4	21	36.2	57	64.8	**0.0021**
	High	37	25.3	22	37.9	15	17.0	
Vitamin supplements	Do not use	42	28.8	11	19.0	31	35.2	**0.0283**
	Periodically	35	24.0	12	20.7	23	26.1	
	Regular	69	47.3	35	60.3	34	38.6	
Mineral supplements	Do not use	74	50.7	26	46.4	48	55.8	0.1154
	Periodically	33	22.6	11	19.6	22	25.6	
	Regular	35	24.0	19	33.9	16	18.6	

TEE, total energy expenditure; BMI, body mass index; BF, body fat; PAL, physical activity level; *n*, number of participants; X, arithmetic mean; SD, standard deviation. The bold values denote statistical significance at the *p* < 0.05 level.

**Table 4:** Comparison of intake levels of selected nutrients from consumed food and supplements between participants according to RapidPOLA index.

Variable		Total *n* = 146		Dietary Fiber in g per kg/m^2^ of BMI ≥ 1 and Mg ≥ 110% Recommendation (NUTRIDIMAF Diet) *n* = 58		Others *n* = 88		U Mann–Whitney Test
		Me (Q1–Q3)		Me (Q1–Q3)		Me (Q1–Q3)		*p*
Energy [kcal]		2136 (1855–2345)		2238 (2025–2549)		2020 (1732–2270)		**<0.0001**
Water [mL]		2497 (2064–3132)		2890 (2455–3449)		2333 (1848–2828)		**<0.0001**
Total protein [g]		77.4 (64.2–96.0)		83 (73–99)		75.1 (60.9–91.7)		**0.0426**
Animal protein [g]		41.6 (29.0–56.2)		38.7 (15.6–52.1)		43.9 (32.3–60.7)		**0.0132**
Plant protein [g]		34.5 (28.6–43.5)		44.8 (33.4–58.6)		30.4 (25.9–36.9)		**<0.0001**
Arginine [mg]		4023 (3235–5244)		4507 (3760–5471)		3674 (2864–4934)		**0.0007**
Fat [g]		69.5 (55.8–82.2)		70 (60.5–88.8)		65.9 (54.7–78.9)		0.0515
Linoleic acid (LA) [g]		9.1 (7.2–12.6)		11.7 (8.7–14.6)		7.9 (6.2–10.4)		**<0.0001**
α-linolenic acid (ALA) [g]		1.6 (1.2–2.2)		1.8 (1.4–2.6)		1.4 (1.1–1.9)		**0.0013**
Omega-6 fatty acids [g]		7.8 (6.3–10.7)		9.7 (8–12.1)		6.6 (5.1–9.2)		**<0.0001**
Omega-3 fatty acids [g]		1.5 (1.1–2.3)		1.8 (1.2–2.5)		1.4 (1.1–2.2)		**0.0410**
Saturated fatty Acid [g]		24.3 (18.1–30.3)		23.1 (18.1–29.8)		24.9 (18.1–30.7)		0.6892
Total carbohydrates [g]		286.9 (244.7–329.0)		310.1 (272.8–374.6)		268.7 (221.9–299.6)		**<0.0001**
Saccharose [g]		41.9 (32.0–55.4)		43.8 (35.8–59.2)		37.6 (28–52.1)		**0.0314**
Dietary fiber [g]		23.9 (18.9–31.6)		33.1 (26.7–40.2)		19.5 (17.5–23.9)		**<0.0001**
Alcohol [g]		5.4 (0–11.7)		5.3 (0–9.9)		5.7 (0–13.3)		0.5202
Potassium [mg]		3542 (2883–4250)		4305 (3646–5092)		3120 (2593–3594)		**<0.0001**
Calcium [mg]		817 (661–1001)		964 (693–1063)		770 (633–908)		**0.0005**
Magnesium [mg]		384 (328–499)		529.5 (404–601.5)		338.7 (298.7–393.3)		**<0.0001**
Iron [mg]		14.6 (12.1–18.4)		18.7 (15–22)		12.7 (10.9–15.4)		**<0.0001**
Zinc [mg]		10.5 (8.8–13.6)		12.7 (10.7–15.2)		9.9 (8.1–11.8)		**<0.0001**
Copper [mg]		1.6 (1.3–2.0)		2.2 (1.7–2.6)		1.3 (1.2–1.6)		**<0.0001**
Manganese [mg]		6.1 (4.4–8.4)		8.4 (6.4–10.9)		4.8 (4–6.4)		**<0.0001**
Vitamin A [µg]		1191 (861–1593)		1419 (1037–1876)		960 (772–1426)		**0.0001**
Beta-carotene [µg]		4336 (2934–6713)		5654 (3833–8710)		3649 (2507–5716)		**<0.0001**
Vitamin E (alpha-tocopherol equivalent) [mg]		11.3 (9.0–15.9)		15.8 (11.7–18.5)		10.2 (8.1–12.5)		**<0.0001**
Thiamin [mg]		1.3 (1.0–1.6)		1.6 (1.3–2.1)		1.1 (0.9–1.4)		**<0.0001**
Riboflavin [mg]		1.7 (1.4–2.1)		1.9 (1.7–2.2)		1.6 (1.3–1.9)		**<0.0001**
Niacin [mg]		17.9 (14.3–24.2)		19 (15.8–24.7)		17.2 (13.8–22.6)		0.0670
Vitamin B6 [mg]		1.9 (1.5–2.5)		2.4 (1.9–3.2)		1.6 (1.4–2)		**<0.0001**
Folates [ µg]		350 (276–455)		469.2 (392–532)		298.8 (247.6–369.3)		**<0.0001**
Vitamin B12 [µg]		3.5 (2.5–5.2)		4.2 (2.9–7.8)		3.1 (2.5–4.5)		**0.0162**
Vitamin C [mg]		114 (72–174)		162.6 (113.6–213.4)		91 (60.4–135)		**<0.0001**
Vitamin D [µg]		3.9 (2,2–13.9)		4 (2.5–30.2)		3.9 (2.1–9)		0.2611
% energy from saturated fatty acids		10.3 (8.4–12.2)		9.45 (7.4–11.7)		10.75 (9.65–12.65)		
Protein [g/kg of body weight]		1.14 (0.98–1.40)		1.2 (1.1–1.5)		1.1 (0.9–1.3)		**0.0011**
Fiber [g per kg/m^2^ of BMI]		1.11 (0.80–1.43)		1.5 (1.2–1.7)		0.9 (0.7–1.1)		**<0.0001**
Variable	Category	*n*	%	*n*	%	*n*	%	Chi^2^ *p*
Protein	<1.2 g/kg b.w.	82	56.2	24	41.4	58	65.9	**0.0035**
	≥1.2 g/kg b.w.	64	43.8	34	58.6	30	34.1	
% energy from SFA	<10	65	44.5	33	56.9	32	36.4	**0.0146**
	≥10	81	55.5	25	43.1	56	63.6	

*n*, number of participants; Me, median; Q1 and Q3, lower and upper quartiles; bold values denote statistical significance at *p* < 0.05.

**Table 5:** Median (Q1–Q3) intake levels of selected nutrients from consumed food and supplements according to RapidPOLA index, expressed as percentage of recommended intake (RDA, AI, or EAR) with between-group comparisons.

Variable	Total *n* = 146	Dietary Fiber in g per kg/m^2^ of BMI ≥ 1 and Mg ≥ 110% Recommendation (NUTRIDIMAF Diet) *n* = 58	Other *n* = 88	U Mann–Whitney Test
	Me (Q1–Q3)	Me (Q1–Q3)	Me (Q1–Q3)	*p*-Value
Water (% of adequate intake)	114.1 (88.2–142.5)	128.2 (104.1–156.9)	99.3 (81.5–124.8)	**<0.0001**
Total protein	121.0 (102.5–143.7)	129.2 (113.1–156.2)	114.2 (97.7–139.8)	**0.0033**
Total fat	78.2 (63.3–94.5)	85.4 (70.3–105.1)	75.4 (60–90)	**0.0036**
Linoleic acid (LA)	80.8 (60.2–106.8)	103.1 (85.8–128.2)	70.6 (53.8–84.4)	<0.0001 *
α-linolenic acid (ALA)	104.5 (79.9–159.0)	126.3 (90.2–177.3)	92.1 (73.3–139.6)	0.0004 *
Assimilable carbohydrates	200.0 (170.2–228.2)	214.6 (189.8–253.2)	189.7 (156.3–213.1)	**<0.0001**
Dietary fiber	95.7 (75.4–126.4)	131.9 (106.8–159.2)	78.1 (70.2–95.7)	<0.0001 *
Potassium	101.2 (82.4–121.4)	123 (104.2–145.5)	89.1 (74.1–102.7)	<0.0001 *
Calcium	81.7 (66.1–100.1)	96.4 (69.3–106.3)	77 (63.3–90.8)	0.0005 *
Magnesium	108.9 (90.6–132.6)	136.1 (123.5–161.5)	93.8 (81.2–104)	<0.0001 *
Iron	115.4 (72.0–168.5)	128.9 (89.6–216.2)	100.2 (69–151.8)	0.0003 *
Zinc	117.2 (95.8–137.5)	134.7 (117.2–158.5)	102.6 (91.3–123.3)	<0.0001 *
Copper	172.4 (142.4–224.4)	246.5 (187.6–293.4)	149.5 (131–180.6)	**<0.0001**
Manganese	290.3 (217.8–396.6)	402.8 (347.2–530.5)	237 (198.1–294.4)	**<0.0001**
Vitamin A	144.0 (106.5–201.4)	174.8 (132.5–240.3)	126.5 (98–172)	0.0001 *
Vitamin E (alpha-tocopherol equivalent)	129.5 (102.1–172.8)	172.1 (138.9–207.1)	112.5 (86.4–133.6)	<0.0001 *
Thiamin	105.1 (85.8–135.6)	133.9 (112.5–161.7)	91.7 (74.1–108)	<0.0001 *
Riboflavin	142.5 (119.7–174.5)	164.9 (135.9–195.3)	131.6 (108.3–155.6)	**<0.0001**
Niacin	119.1 (97.0–159.5)	130.4 (107.5–168.5)	113 (90.8–153.4)	**0.0430**
Vitamin B6	147.8 (116.8–188.8)	187.8 (149.3–249.4)	125.6 (110.2–156.7)	<0.0001 *
Folates	87.5 (69.1–113.8)	117.3 (98–133)	74.7 (61.9–92.3)	<0.0001 *
Vitamin B12	145.6 (106.2–226.9)	184.8 (122.3–323.2)	128.8 (103.2–187.4)	**0.0069**
Vitamin C	142.0 (91.7–227.7)	212.8 (138.3–247.7)	105.2 (72–159.5)	<0.0001 *
Vitamin D	26.0 (14.9–92.6)	26.7 (16.9–201.3)	25.7 (14–59.7)	0.2611 *

*n*, number of participants; Me, median; Q1 and Q3, lower and upper quartiles; the bold values denote statistical significance at the *p* < 0.05 level; * component included in the POLA index.

**Table 6:** The consumption of selected foods according to the RapidPOLA index. The continuous variables are presented as the median (Q1–Q3) intake in grams per day; the categorical variables represent the proportion of the participants meeting the predefined intake thresholds.

Variable		Total *n* = 146		Dietary Fiber in g per kg/m^2^ of BMI ≥ 1 and Mg ≥ 110% Recommendation (NUTRIDIMAF Diet) *n* = 58		Other *n* = 88		U Mann–Whitney Test
		Me (Q1–Q3)		Me (Q1–Q3)		Me (Q1–Q3)		*p*-Value
Bread [g/day]		106.6 (66.7–138.1)		99.5 (62.9–138.1)		113.3 (66.7–138.3)		0.7884
Groats and rice [g/day]		14.0 (5.1–28.2)		17.5 (8.7–32.1)		10.6 (1.7–24.6)		**0.0160**
Seeds [g/day]		1.7 (0–5.9)		4.6 (1.7–12.5)		0.8 (0–2.6)		**<0.0001**
Nuts [g/day]		13.1 (2.2–26.9)		20.6 (11.5–36)		8.6 (1.5–17.3)		**0.0001**
Seeds and nuts [g/day]		17.3 (4.7–36.8)		31.9 (17.5–47.9)		10.9 (3–21.1)		**<0.0001**
Fruit [g/day]		206.1 (124.5–339.4)		280.3 (172.6–430.8)		155 (99.5–280)		**<0.0001**
Vegetables [g/day]		331.2 (242.8–446.0)		420.8 (331.1–529.3)		267.7 (193.5–377.1)		**<0.0001**
Total vegetables and fruit (in market products) [g/day]		557.1 (413.2–738.3)		680.2 (579.1–917.4)		464.1 (362.9–630.2)		**<0.0001**
Potatoes [g/day]		116.6 (74.1–171.4)		130.3 (86.1–192.9)		103.9 (67.5–161.8)		0.0517
Legumes [g/day]		6.8 (0–37.4)		30.3 (4.8–94.4)		2.7 (0–17)		**<0.0001**
Oils [g/day]		9.6 (4.6–16.8)		12.4 (7.5–19.3)		8.3 (4–13.6)		**0.0042**
Butter [g/day]		5.3 (1.4–12.4)		2 (0–6)		2.5 (0.7–11.2)		0.2259
Milk [g/day]		71.3 (21–144.8)		41.5 (11.9–136.7)		73.1 (27.6–148.4)		0.0955
Fermented milk drinks [g/day]		13.3 (0–60.8)		4.7 (0–60.8)		23.2 (0–64.3)		0.2818
Hard cheese [g/day]		16.8 (6.7–32.5)		13.1 (0.7–27.9)		20.1 (8.7–35)		0.0562
Eggs [g/day]		46.3 (19.5–73.2)		46.2 (16.8–85.5)		46.3 (21.7–67.1)		0.8717
Meat, cold cuts and poultry [g/day]		94.9 (20.2–175.7)		32.9 (5.5–127.2)		112 (47.5–190.4)		**0.0017**
Fish and seafood [g/day]		11.7 (0–33.3)		1.3 (0–22.8)		13.3 (0–37.3)		0.1100
Variable	Category	*n*	%	*n*	%	*n*	%	Chi^2^ *p*
Fruits and vegetables	<400 g/day	33	22.6	2	3.4	31	35.2	<0.0001 *
	400–<600 g/day	45	30.8	13	22.4	32	36.4	
	≥600 g/day	68	46.6	43	74.1	25	28.4	
Nuts	≥10 g/day	86	58.9	44	75.9	42	47.7	0.0007 *
	<10 g/day	60	41.1	14	24.1	46	52.3	

*n*, number of participants; Me, median; Q1 and Q3, lower and upper quartiles; the bold values denote statistical significance at the *p* < 0.05 level; * component included in the POLA index.

**Table 7:** Odds ratios for contracting COVID-19 or influenza during follow-up according to POLA and RapidPOLA indexes.

Model		OR (95% CI)
**POLA index**	**BIM**	**UBIM + HUBIM**
Model 1 ^a^	1 (ref.)	3.8 (1.48–9.45)
Model 2 ^b^	1 (ref.)	3.0 (1.11–8.07)
**RapidPOLA index**	**Dietary fiber in g per kg/m^2^ of BMI ≥ 1** **and Mg ≥ 110% recommendation (NUTRIDIMAF diet)**	**Other**
Model 1 ^a^	1 (ref.)	4.1 (1.69–10.26)
Model 2 ^b^	1 (ref.)	3.4 (1.28–8.75)

^a^ crude model; ^b^ adjusted to type of diet, sex, marital status, physical activity, year of cohort, and WHtR, OR—odds ratio.

**Table 8:** The correlation between dietary fiber in g per kg/m^2^ and the recommended magnesium %, and components included in the POLA index.

		Dietary Fiber in g per kg/m^2^ of BMI	Magnesium [% Recommended]
POLA index	r	−0.80	−0.79
	*p*	**<0.0001**	**<0.0001**
Potassium [% recommended]	r	0.68	0.69
	*p*	**<0.0001**	**<0.0001**
Calcium [% recommended]	r	0.23	0.36
	*p*	**0.0061**	**<0.0001**
Iron [% recommended]	r	0.45	0.35
	*p*	**<0.0001**	**<0.0001**
Zinc [% recommended]	r	0.51	0.65
	*p*	**<0.0001**	**<0.0001**
Vitamin A [% recommended]	r	0.38	0.33
	*p*	**<0.0001**	**<0.0001**
Vitamin E [% recommended]	r	0.58	0.59
	*p*	**<0.0001**	**<0.0001**
Thiamin [% recommended]	r	0.61	0.58
	*p*	**<0.0001**	**<0.0001**
Riboflavin [% recommended]	r	0.32	0.40
	*p*	**<0.0001**	**<0.0001**
Niacin [% recommended]	r	0.10	0.22
	*p*	0.2440	0.0078
Vitamin B6 [% recommended]	r	0.54	0.54
	*p*	**<0.0001**	**<0.0001**
Vitamin C [% recommended]	r	0.55	0.45
	*p*	**<0.0001**	**<0.0001**
Linoleic acid (LA) [% recommended]	r	0.53	0.56
	*p*	**<0.0001**	**<0.0001**
α-Linolenic acid (ALA) [% recommended]	r	0.42	0.33
	*p*	**<0.0001**	**<0.0001**
Folates [% recommended]	r	0.76	0.65
	*p*	**<0.0001**	**<0.0001**
Vitamin B12 [% recommended]	r	0.17	0.25
	*p*	**0.0419**	**0.0020**
Vitamin D [% recommended]	r	0.09	0.10
	*p*	0.2661	0.2387
Nuts [g/day]	r	0.36	0.45
	*p*	**<0.0001**	**<0.0001**
Total vegetables and fruit (in market products) [g/day]	r	0.67	0.49
	*p*	**<0.0001**	**<0.0001**

r—Spearman correlation; the bold values denote statistical significance at the *p* < 0.05 level.

**Table 9:** Comparison of dietary intake between type of diet consumed for participants with ≥1 g fiber per kg/m^2^ of BMI and ≥110% of recommended magnesium intake (NUTRIDIMAF diet).

Variable		Traditional *n* = 26		Vegetarian *n* = 32		U Mann–Whitney Test
		Me (Q1–Q3)		Me (Q1–Q3)		*p*
Energy [kcal]		2210 (1952–2549)		2263 (2087–2479)		0.8025
Water [mL]		3022 (2634–3870)		2723 (2327–3188)		0.0650
Total protein [g]		88.8 (76.7–101.4)		76.6 (70.3–94)		**0.0421**
Animal protein [g]		52.7 (42.7–59.6)		22.5 (3.2–37.1)		**<0.0001**
Plant protein [g]		35.1 (31.8–42.9)		55.7 (44.9–67.9)		**<0.0001**
Arginine [mg]		4389 (3760–5523)		4625 (3754–5341)		0.9501
Fat [g]		76.5 (64.4–92.5)		69.5 (56.2–84.9)		0.1108
Linoleic acid (LA) [g]		10.8 (8.2–13.2)		13.1 (9.7–15.6)		**0.0286**
α-linolenic acid (ALA) [g]		1.5 (1.1–2.1)		2.2 (1.6–3.2)		**0.0044**
Omega-6 fatty acids [g]		9.5 (8.3–11)		10.2 (7.9–12.4)		0.6168
Omega-3 fatty acids [g]		9.5 (8.3–11)		10.2 (7.9–12.4)		0.3813
Saturated fatty Acid [g]		26.2 (21.5–32.5)		19.5 (16.1–26.6)		**0.0036**
Total carbohydrates [g]		304.6 (267.3–373.3)		328 (291.3–376.6)		0.1416
Saccharose [g]		50.8 (35.8–62.8)		42.4 (35.2–57.4)		0.3095
Dietary fiber [g]		31 (25.3–33.2)		37.2 (30.8–46.2)		**0.0024**
Alcohol [g]		4.6 (0–8.6)		5.4 (0.5–13.7)		0.3636
Potassium [mg]		4280 (3646–5123)		4321 (3690–5041)		0.7545
Calcium [mg]		981.9 (693.2–1041.7)		905.6 (741.1–1101.9)		0.9005
Magnesium [mg]		439.9 (395.2–545.7)		567.3 (503–651.6)		**0.0094**
Iron [mg]		18.1 (13.6–20.7)		20.1 (17–23)		0.0975
Zinc [mg]		13 (10.7–17.4)		12.7 (10.6–14.4)		0.5215
Vitamin A [µg]		1540 (1248–2072)		1319 (925–1700)		**0.0310**
Beta-carotene [µg]		5472 (4659–9035)		5818 (3236–7902)		0.6729
Vitamin E (alpha-tocopherol equivalent) [mg]		16.1 (12.2–18.7)		14.6 (11.2–18.1)		0.4530
Thiamin [mg]		1.6 (1.3–1.9)		1.6 (1.3–2.1)		0.7427
Riboflavin [mg]		2.1 (1.8–2.7)		1.8 (1.7–2)		**0.0154**
Niacin [mg]		23.6 (19.4–28.6)		16.8 (14.3–20.1)		**0.0003**
Vitamin B6 [mg]		2.8 (2.2–3.3)		2.4 (1.9–2.9)		0.0799
Folates [ µg]		456.2 (347.7–514.2)		477.9 (417.8–598.6)		0.1839
Vitamin B12 [µg]		4.4 (3.2–6.1)		3.7 (2.1–21.5)		0.7545
Vitamin C [mg]		183.1 (157.8–243.5)		141.3 (106.5–178.6)		**0.0264**
Vitamin D [µg]		8.7 (3.5–49.2)		2.8 (1.6–11.7)		**0.0012**
POLA index score [points]		4.5 (2–6)		4 (3–5.5)		0.8933
% energy from saturated fatty acids		10.9 (8.9–12.5)		8.4 (6.3–9.9)		**0.0011**
Protein [g/kg of body weight]		1.36 (1.24–1.66)		1.13 (0.97–1.36)		**0.0012**
Variable	Category	*n*	%	*n*	%	Chi^2^ *p*
Protein	<1.2 g/kg b.w.	6	23.1	18	56.3	**0.0107**
	≥1.2 g/kg b.w.	20	76.9	14	43.8	
% energy from SFA	<10	9	34.6	24	75.0	**0.0020**
	≥10	17	65.4	8	25.0	

*n*, number of participants; Me, median; Q1 and Q3, lower and upper quartiles; bold values denote statistical significance at *p* < 0.05.

**Table 10:** The consumption of selected foods according to the type of diet for the participants in the ≥1 g fiber per kg/m^2^ of BMI and ≥110% recommended magnesium intake (NUTRIDIMAF diet) group. The continuous variables are presented as the median (Q1–Q3) intake in grams per day; the categorical variables represent the proportion of the participants meeting the predefined intake thresholds.

Variable		Traditional *n* = 26		Vegetarian *n* = 32		U Mann–Whitney Test
		Me (Q1–Q3)		Me (Q1–Q3)		*p*-Value
Bread [g/day]		115.2 (73–138.1)		98 (56.4–145.5)		0.6059
Groats and rice [g/day]		14.8 (4.3–26.5)		25.3 (13.1–52.1)		**0.0198**
Seeds [g/day]		3.3 (1.4–6)		7 (2.4–19)		**0.0210**
Nuts [g/day]		20.2 (6–34.4)		20.9 (11.5–39.7)		0.5629
Seeds and nuts [g/day]		26.3 (11.5–45.8)		36.8 (22.9–60.5)		0.1416
Fruit [g/day]		297.2 (182.9–501.5)		269.3 (166.2–413.6)		0.2347
Vegetables [g/day]		427 (328.6–515.1)		407.8 (337.2–566.5)		0.5950
Total vegetables and fruit (in market products) [g/day]		735.5 (634–986.9)		634.5 (564.2–901.5)		0.5735
Potatoes [g/day]		134.7 (94.5–211)		117.3 (82.4–166.1)		0.1548
Legumes [g/day]		5.3 (0–11.5)		89.3 (42.3–136)		**<0.0001**
Oils [g/day]		12.8 (7.8–19.3)		11.8 (6.8–18.8)		0.5950
Butter [g/day]		3.1 (1.7–11.2)		0.5 (0–4)		**0.0252**
Milk [g/day]		107.3 (33.5–243.8)		24.5 (3.5–71.4)		**0.0008**
Fermented milk drinks [g/day]		14.9 (0.1–66.7)		0 (0–47)		0.0693
Hard cheese [g/day]		15.3 (6–29.5)		9.4 (0–26.4)		0.2290
Eggs [g/day]		64.4 (29.4–98.4)		32.1 (7.8–63.2)		**0.0103**
Meat, cold cuts and poultry [g/day]		127.8 (102–191.3)		5.9 (0.8–21.3)		**<0.0001**
Fish and seafood [g/day]		17 (6–34)		0 (0–1.3)		**0.0002**
Variable	Category	*n*	%	*n*	%	Chi^2^ *p*
Fruits and vegetables	<400 g/day	1	3.8	1	3.1	0.5117 *
	400–<600 g/day	4	15.4	9	28.1	
	≥600 g/day	21	80.8	22	68.8	
Nuts	≥10 g/day	19	73.1	25	78.1	0.6550 *
	<10 g/day	7	26.9	7	21.9	

*n*; number of participants; Me, median; Q1 and Q3, lower and upper quartiles; the bold values denote statistical significance at the *p* < 0.05 level; * component included in the POLA index.

## Data Availability

The data presented in this study are not publicly available due to confidentiality reasons. These data are available on request from the corresponding author.

## Supplementary Materials

The following supporting information can be downloaded at: https://www.mdpi.com/article/10.3390/nu18111689/s1, Table S1. Characteristics of participants according to StrongPOLA groups. Table S2. Comparison of dietary intake between participants according to StrongPOLA groups. Table S3. Median (Q1–Q3) intake levels of selected nutrients according to StrongPOLA groups, expressed as percentage of recommended intake (RDA, AI, or EAR) with between-group comparisons. Table S4. The consumption of selected food products according to the StrongPOLA groups. The continuous variables are presented as the median (Q1–Q3) intake in grams per day; the categorical variables represent the proportion of the participants meeting the predefined intake thresholds.

## Abbreviations

The following abbreviations are used in this manuscript:

BIM	Beneficial Immunomodulation (POLA category ≤ 5)
CI	Confidence Interval
HUBIM	Highly Unbeneficial Immunomodulation (POLA category ≥ 12)
OR	Odds Ratio
PAL	Physical Activity Level
TEE	Total Energy Expenditure
UBIM	Unbeneficial Immunomodulation (POLA category 6–11)
WHtR	Waist-to-height ratio
