# Supplementary material for: Interactive Tree Analysis Identifies Dietary Fiber and Magnesium Adequacy as Exploratory Screening Markers for Assessing Nutrient-Dense, Immune-Supportive and Anti-Inflammatory Dietary Patterns in Young Adults Without Comorbidities: Proposition of the New StrongPOLA and RapidPOLA Indexes
Source: Nutrients. 2026 May 25;18(11):1689. doi: 10.3390/nu18111689 (PMC13257699; doi:10.3390/nu18111689)
Supplement: Supplementary file 1 [file nutrients-18-01689-s001.zip › nutrients-4251943-supplementary.pdf]

nutrients

MDPI

Table S1. Characteristics of participants according to StrongPOLA group.

Variable		Dietary fiber in g per kg/m² of BMI ≥ 1 and Mg >130% recommendation (strongNUTRI DIMAF diet) *n*=37		Dietary fiber in g per kg/m² of BMI ≥ 1 and Mg <130% recommendation *n*=49		Dietary fiber in g per kg/m² of BMI < 1 *n*=60		*ANOVA* p
		X ± SD		X ± SD		X ± SD		
Age [years]		33.0± 5.2		35.7± 5.9		35.4± 5.4		0.0528
Height [cm]		176.9± 10		173.3± 9.2		171.2± 8.5		**0.0130**
Body weight [kg]		70.9± 12.8		66.9± 11.9		70.6± 11.6		0.1980
BMI [kg/m²]		22.5± 2.9		22.1± 2.3		24± 2.5		**0.0005**
WHtR		0.46± 0.04		0.47± 0.04		0.49± 0.05		**0.0050**
TEE [kcal]		2529± 512		2360± 442		2402± 421		0.2186
PAL		1.51± 0.22		1.49± 0.14		1.49± 0.14		0.8201
Sleep duration [h]		7.39±0.53		7.20±0.46		7.24±0.46		0.1777
Steps		12908± 5684		12630± 3856		12387± 4045		0.8542
POLA index [points]		3.4± 2		7.7± 3		12.4± 3.3		**<0.0001**
**Variable**	**Category**	*n*	%	*n*	%	*n*	%	**Chi² p**
Sex	Men	22	59.5	23	46.9	28	46.7	0.4118
	Women	15	40.5	26	53.1	32	53.3	
Diet type	Traditional	14	37.8	27	55.1	49	81.7	**<0.0001**
	Vegetarian	23	62.2	22	44.9	11	18.3	
BMI [kg/m²]	Normal body weight	29	78.4	42	85.7	37	61.7	**0.0136**
	Overweight	8	21.6	7	14.3	23	38.3	
Marital status	Single/divorced	27	73.0	23	46.9	23	38.3	**0.0036**
	Married/cohabiting	10	27.0	26	53.1	37	61.7	
BF [%]	Underfat or Normal	33	89.2	43	89.6	43	71.7	**0.0232**
	Overfat	4	10.8	5	10.4	17	28.3	
Smoking status	No	35	94.6	43	87.8	50	83.3	0.2611
	Yes	2	5.4	6	12.2	10	16.7	
Education level	Secondary	4	10.8	3	6.1	2	3.3	0.3308
	Higher	33	89.2	46	93.9	58	96.7	
Self-rated physical activity (work)	Low	29	78.4	40	81.6	40	66.7	0.2777
	Moderate	8	21.6	7	14.3	17	28.3	
	High	0	0	2	4.1	3	5.0	
Self-rated physical activity (leisure)	Low	10	27.0	10	20.4	11	18.3	**0.0316**
	Moderate	12	32.4	27	55.1	39	65.0	
	High	15	40.5	12	24.5	10	16.7	
	I don't use	6	16.2	14	28.6	22	36.7	0.1007
	Periodically	7	18.9	12	24.5	16	26.7	

Vitamin supplementation	Regular	24	64.9	23	46.9	22	36.7	
Supplementation with minerals	I don't use	16	45.7	28	57.1	30	51.7	
	Periodically	6	17.1	12	24.5	15	25.9	0.3549
	Regular	13	37.1	9	18.4	13	22.4	

TEE—total energy expenditure; BMI—body mass index; BF—body fat; PAL—physical activity level; *n*—number of participants; *x*X —arithmetic mean; SD—standard deviation. Bold values denote statistical significance at the p < 0.05 level.

Table S2. Comparison of intake levels of selected nutrients from consumed food and supplements between participants according to StrongPOLA group.

Variable	Dietary fiber in g per kg/m² of BMI ≥ 1 and Mg ≥130% recommendation (strongNUTRIDIMAF diet) *n*=37	Dietary fiber in g per kg/m² of BMI ≥ 1 and Mg <130% recommendation *n*=49	Dietary fiber in g per kg/m² of BMI < 1 *n*=60	ANOVA Kruskal-Wallis
	**Me (Q1 – Q3)**	**Me (Q1 – Q3)**	**Me (Q1 – Q3)**	**p**
Energy [kcal]	2341 (2183-2634)	2145 (1855-2274)	2002 (1626-2203)	**< 0.0001**
Water [ml]	3108 (2540-3561)	2511 (2307-3063)	2193 (1686-2648)	**< 0.0001**
Total protein [g]	90 (74.3-100.6)	77.1 (62.6-90.8)	75.5 (60.9-90)	**0.0236**
Animal protein [g]	36.8 (5.5-49.4)	38.6 (26.9-54.9)	45.8 (34.7-57.9)	**0.0133**
Plant protein [g]	53 (42.9-65.4)	36.6 (32.3-41.5)	28.3 (24-33.1)	**< 0.0001**
Arginine [mg]	5181 (4306-5598)	3731 (3152-5094)	3699 (2861-4751)	**< 0.0001**
Fat [g]	74.5 (64.4-90.1)	67.4 (54-85)	65.7 (54.7-74.8)	**0.0152**
Linoleic acid LA (C18:2) [g]	14 (11.2-15.7)	8.9 (7.5-11.2)	7.3 (6-9.5)	**< 0.0001**
α-Linolenic acid ALA (C18:3) [g]	2.2 (1.5-3.2)	1.7 (1.2-2.2)	1.2 (1.1-1.7)	**< 0.0001**
Omega−3 fatty acids [g]	2.2 (1.3-2.7)	1.7 (1.2-2.2)	1.3 (1-1.9)	**0.0048**
Omega−6 fatty acids [g]	10.9 (8.7-13.1)	8.1 (6.7-10)	6.4 (4.8-7.7)	**< 0.0001**
Saturated Fatty Acid [g]	22.3 (17.6-30.3)	24.7 (17.9-31.5)	24.9 (19.1-30.1)	0.8286
Total carbohydrates [g]	347.5 (307.9-390.4)	290.2 (267.3-320)	248.3 (212.6-285.6)	**< 0.0001**
Saccharose [g]	51.3 (39.8-61.5)	36.3 (32.2-45.7)	40.5 (26.4-55.5)	**0.0065**
Dietary fiber [g]	37.6 (33.2-46)	25.8 (23.6-29.2)	18.3 (16.4-19.7)	**< 0.0001**
Alcohol [g]	6 (1.1-11.5)	5.3 (0.4-10.1)	5.3 (0-13.8)	0.7886
Potassium [mg]	4673 (4125-5531)	3572 (3119-3841)	2945 (2498-3457)	**< 0.0001**
Calcium [mg]	924.2 (761.8-1090.8)	857.1 (684.5-1004.5)	766.7 (633-914.5)	**0.0286**
Magnesium [mg]	581.2 (542.8-660.5)	383.7 (352.8-412.2)	326.5 (284.9-370)	**< 0.0001**
Iron [mg]	20.8 (18.6-22.9)	13.9 (12.7-16.3)	12.4 (10.5-14.5)	**< 0.0001**
Zinc [mg]	14.3 (12.4-17.3)	10.1 (9.2-13.1)	9.6 (7.8-10.8)	**< 0.0001**
Copper [mg]	2.4 (2.1-2.9)	1.6 (1.4-1.8)	1.3 (1.1-1.5)	**< 0.0001**
Manganese [mg]	9.2 (8.3-11.5)	6.4 (5-7.1)	4.3 (3.6-5.3)	**< 0.0001**

https://doi.org/10.3390/nu18111689

Vitamin A [μg]	1447 (1178-1929)	1332 (922-1626)	953 (754-1211)	**< 0.0001**
Beta-carotene [μg]	6481 (4212-9035)	5205 (3517-6988)	3077 (2168-4536)	**< 0.0001**
Vitamin E (alpha-tocopherol equivalent) [mg]	16.2 (13.9-19.2)	11.5 (9.8-14.5)	9.1 (7.1-11)	**< 0.0001**
Thiamin [mg]	1.7 (1.5-2.2)	1.3 (1.1-1.5)	1 (0.8-1.3)	**< 0.0001**
Riboflavin [mg]	1.9 (1.7-2.2)	1.8 (1.4-2.1)	1.6 (1.3-1.8)	**0.0007**
Niacin [mg]	21.1 (16.1-28.6)	17.7 (13.8-22.8)	17.5 (14.3-22)	0.0879
Vitamin B6 [mg]	2.8 (2.4-3.3)	1.8 (1.5-2.3)	1.6 (1.4-2)	**< 0.0001**
Folates [μg]	495.5 (461.6-557.6)	361.2 (331.9-417.6)	272.5 (227.3-322.5)	**< 0.0001**
Vitamin B12 [μg]	4.4 (2.5-9.1)	3.5 (2.5-5.2)	3.1 (2.5-4.1)	0.1125
Vitamin C [mg]	162.8 (119.6-210.8)	137.9 (92.3-193.8)	70.6 (49.4-115.3)	**< 0.0001**
Vitamin D [μg]	3.7 (2.5-30.2)	3.8 (2.4-22.7)	4.1 (2-7.8)	0.5143
% energy from Saturated fatty Acid	8.7 (7.1-10.1)	10.6 (8.2-12.2)	10.9 (9.75-13)	**0.0001**
Protein [g/kg of body weight]	1.24 (1.07-1.49)	1.14 (0.97-1.41)	1.06 (0.93-1.25)	**0.0199**
Fiber [g per kg/m^2 of BMI]	1.66 (1.54-1.89)	1.16 (1.1-1.26)	0.75 (0.67-0.86)	**< 0.0001**

**Variable**	**Category**	***n***	**%**	***n***	**%**	***n***	**%**	**Chi^2 p**
Protein	< 1.2 g/kg b.w.	16	43.2	26	53.1	40	66.7	0.0676
	≥ 1.2 g/kg b.w.	21	56.8	23	46.9	20	33.3	
% energy from SFA	< 10	24	64.9	23	46.9	18	30.0	**0.0033**
	⩾ 10	13	35.1	26	53.1	42	70.0	

n – number of participants, Me - median, Q1 and Q3 – lower and upper quartile, bold values denote statistical significance at the *p* <0,05

Table S3. Median (Q1–Q3) intake levels of selected nutrients from consumed food and supplements according to StrongPOLA group, expressed as percentage of recommended intake (RDA, AI, or EAR), with between-group comparisons.

**Variable**	**Dietary fiber in g per kg/m^2 of BMI ≥ 1 and Mg ≥130% recommendation (strongNUTRIDIMAF diet) *n*=37**	**Dietary fiber in g per kg/m^2 of BMI ≥ 1 and Mg <130% recommendation *n*=49**	**Dietary fiber in g per kg/m^2 of BMI < 1 *n*=60**	**ANOVA Kruskal-Wallis**
	**Me (Q1 – Q3)**	**Me (Q1 – Q3)**	**Me (Q1 – Q3)**	**p**
Water (% of adequate intake)	128.1 (117.4-157.5)	116.7 (98.2-137.3)	93.9 (75.2-124.3)	**0.0001**
Total protein	129.5 (116.3-153.8)	123.9 (103.6-150.2)	114.4 (97.7-132.8)	**0.0263**
Total fat	88.1 (71.9-100.3)	77.1 (62-97.7)	72.9 (60.8-89)	**0.0276**
Linoleic acid LA (C18:2)	114.3 (94.5-143.9)	82.8 (72.6-94.5)	62.1 (52.1-79.2)	**< 0.0001***
α-Linolenic acid ALA (C18:3)	137.6 (104.3-213)	122 (81-164.6)	88 (72.2-109.9)	**< 0.0001***

https://doi.org/10.3390/nu18111689

Assimilable carbohydrates	239.3 (202.8-266.1)	204.3 (185.8-222.2)	176 (150.3-203.8)	**< 0.0001**
Dietary fiber	148.8 (132.3-182.7)	103.3 (94.5-116.6)	73.2 (65.5-78.8)	**< 0.0001***
Potassium	133.5 (117.9-158)	102.1 (89.1-109.7)	84.1 (71.4-98.8)	**< 0.0001***
Calcium	92.4 (76.2-109.1)	85.7 (68.5-100.5)	76.7 (63.3-91.4)	**0.0286***
Magnesium	157.3 (137-177)	108.8 (97.4-118.1)	89 (76.5-103.2)	**< 0.0001***
Iron	183.2 (113.8-226.3)	121.2 (71.8-161)	90.3 (65.3-126.6)	**< 0.0001***
Zinc	145 (129.4-175.2)	117.8 (101.8-128)	96.9 (91-118.7)	**< 0.0001***
Copper	270.4 (238.8-318.3)	180.3 (157.2-204.1)	139.7 (123.2-161.1)	**< 0.0001**
Manganese	478.5 (378.4-572.6)	301.7 (246.5-370.7)	217.4 (186.4-273.4)	**< 0.0001**
Vitamin A	194.3 (134.3-240.3)	166.2 (118.6-205.2)	116.6 (96.2-145.9)	**0.0001***
Vitamin E (alpha-tocopherol equivalent)	176.8 (156.9-217.9)	128.9 (112.1-152.2)	102.3 (80.4-130.7)	**< 0.0001***
Thiamin	139.6 (122.1-178.7)	105.2 (92.9-127.9)	86.7 (73.3-104)	**< 0.0001***
Riboflavin	165.6 (132.5-195.3)	149.5 (119.7-178.1)	129.9 (112.8-153)	**0.0011**
Niacin	133.3 (107.5-178.5)	113.6 (88-157.9)	118.1 (95-154.1)	0.1257
Vitamin B6	213.3 (180.9-251)	142.1 (118.8-175.6)	124.2 (107.9-151.1)	**< 0.0001***
Folates	123.9 (115.4-139.4)	90.3 (83-104.4)	68.1 (56.8-80.6)	**< 0.0001***
Vitamin B12	186.4 (122.2-380.9)	146 (103-216.6)	127.2 (106.2-170.3)	0.0518
Vitamin C	210.5 (151.7-243.4)	153.2 (113.4-254.2)	92.7 (63-143.3)	**< 0.0001***
Vitamin D	24.4 (16.9-201.3)	25.6 (16.1-151)	27.4 (13.4-52.1)	0.5131*

n - number of participants, Me—median, Q1 and Q3—lower and upper quartile, bold values denote statistical significance at the p < 0.05 level, * component included in the POLA index

Table S4. Consumption of selected food products according to StrongPOLA group. Continuous variables are presented as median (Q1–Q3) intake in grams per day; categorical variables represent the proportion of participants meeting predefined intake thresholds.

**Variable**		**Dietary fiber in g per kg/m² of BMI ≥ 1 and Mg ≥130% recommendation (strongNUTRIDIM AF diet)** *n*=37		**Dietary fiber in g per kg/m² of BMI ≥ 1 and Mg <130% recommendation** *n*=49		**Dietary fiber in g per kg/m² of BMI < 1** *n*=60		**ANOVA Kruskal-Wallis**
		**Me (Q1 – Q3)**		**Me (Q1 – Q3)**		**Me (Q1 – Q3)**		***p* - value**
Groats and rice [g/day]		21.2 (10.2-38.5)		14.6 (6.5-27.1)		10.2 (1-22.4)		**0.0163**
Seeds[g/day]		4.6 (2.8-16.4)		2.3 (0.1-8.4)		0.1 (0-1.9)		**<0.0001**
Nuts [g/day]		25 (17.3-42.4)		12.9 (2.7-22.8)		6.7 (0.5-17.1)		**<0.0001**
Seeds and nuts [g/day]		37.3 (25.9-60.6)		17.2 (6-29.7)		9.3 (1.2-19.3)		**<0.0001**
Fruit [g/day]		259.1 (172.6-440.7)		263.9 (152.1-341.7)		142.2 (89.2-235.6)		**0.0001**
Vegetables [g/day]		439 (360.5-572.5)		391.2 (303.3-484.2)		241 (169.7-314.9)		**<0.0001**
Total vegetables and fruit (in market products) [g/day]		663.4 (603.5-960.5)		666.3 (510-781.8)		409.4 (293.8-538)		**<0.0001**
Legumes [g/day]		53.5 (8-113.9)		8.7 (0-27.6)		1.5 (0-10.4)		**<0.0001**
**Variable**	**Category**	**N**	**%**	**N**	**%**	**N**	**%**	**Chi²**

								p
Fruit and vegatable	<400 g/day	1	2.7	3	6.1	29	48.3	**<0.0001***
	400–<600 g/day	8	21.6	17	34.7	20	33.3	
	≥600 g/day	28	75.7	29	59.2	11	18.3	
Nuts	10 g and more per day	32	86.5	29	59.2	25	41.7	**<0.0001***
	Up to 10 g per day	5	13.5	20	40.8	35	58.3	

n—number of participants, Me—median, Q1 and Q3—lower and upper quartile, bold values denote statistical significance at the $p < 0.05$ level, * component included in the POLA index

Disclaimer/Publisher's Note: The statements, opinions and data contained in all publications are solely those of the individual author(s) and contributor(s) and not of MDPI and/or the editor(s). MDPI and/or the editor(s) disclaim responsibility for any injury to people or property resulting from any ideas, methods, instructions or products referred to in the content.
